# Screening for the Active Anti-Inflammatory and Antioxidant Polyphenols of *Gaultheria procumbens* and Their Application for Standardisation: From Identification through Cellular Studies to Quantitative Determination

**DOI:** 10.3390/ijms222111532

**Published:** 2021-10-26

**Authors:** Monika Anna Olszewska, Aleksandra Owczarek, Anna Magiera, Sebastian Granica, Piotr Michel

**Affiliations:** 1 Department of Pharmacognosy, Faculty of Pharmacy, Medical University of Lodz, Muszynskiego 1 St., 90-151 Lodz, Poland; monika.olszewska@umed.lodz.pl (M.A.O.); aleksandra.owczarek@umed.lodz.pl (A.O.); anna.magiera@umed.lodz.pl (A.M.); 2 Microbiota Lab, Centre for Preclinical Studies, Department of Pharmacognosy and Molecular Basis of Phytotherapy, Medical University of Warsaw, 1 Banacha St., 02-097 Warsaw, Poland; sgranica@wum.edu.pl

**Keywords:** *Gaultheria procumbens*, anti-inflammatory activity, antioxidant activity, isolation, structure elucidation, quercetin 3-*O*-*β*-d-xylopyranosyl-(1→2)-*β*-d-glucuronopyranoside, miquelianin potassium salt, gaultherin, cinnamtannin B-1, standardisation

## Abstract

Aerial parts, leaves, and stems of *Gaultheria procumbens* are polyphenol-rich herbal medicines with anti-inflammatory and antioxidant effects. The present study focused on identifying active markers of the *G. procumbens* extracts in an integrated approach combining phytochemical and biological capacity tests. The target compounds, representing all classes of *Gaultheria* polyphenols, were pre-selected by LC-ESI-PDA-MS/MS. For unambiguous identification, the key analytes, including a rare procyanidin trimer (cinnamtannin B-1), miquelianin potassium salt, and two new natural products: quercetin and kaempferol 3-*O*-*β*-d-xylopyranosyl-(1→2)-*β*-d-glucuronopyranosides, were isolated by preparative HPLC and investigated by spectroscopy (HR-ESI-MS, UV-vis, CD, 1D- and 2D-NMR), thiolysis, flame photometry, optical rotation experiments, and absolute configuration studies. The significant contribution of the pre-selected compounds to the biological effects of the extracts was confirmed in vitro: the analytes significantly and in a dose-dependent manner down-regulated the pro-oxidant and pro-inflammatory functions of human neutrophils ex vivo (inhibited the release of reactive oxygen species, IL-1β, TNF-α, and neutrophils elastase, ELA-2), inhibited two key pro-inflammatory enzymes (cyclooxygenase, COX-2, and hyaluronidase), and most of them, except gaultherin, exerted potent direct antioxidant activity (ferric reducing antioxidant power and superoxide anion scavenging capacity). Moreover, cellular safety was confirmed for all compounds by flow cytometry. Eventually, as these mechanisms have been connected to the health benefits of *G. procumbens*, 11 polyphenols were accepted as active markers, and a simple, accurate, reproducible, and fully validated RP-HPLC-PDA method for standardisation of the target extracts was proposed.

## 1. Introduction

Quality control (QC) is the main factor in guaranteeing the therapeutic effectiveness and safety of herbal medicines [1]. However, plant-based products are usually complex mixtures with no apparent targets for standardisation [2]. The most profitable strategy for selecting QC markers is based on the biological potency of the individual compounds and their contribution to the pharmacological effects of herbal products [3]. On the other hand, two sets of analytical procedures are recommended for the QC process: chromatographic fingerprinting with mass spectral (MS) detection (for authentication purposes) and specific quantitative assays of selected phytochemicals that drive therapeutic properties [4,5]. Considering the molecular diversity of plant constituents, proper structural annotation of fingerprints is still challenging despite the advantages of multistage mass spectral (MS*^n^*) profiling [6]. In the case of rare phytochemicals, especially glycosides, identification of their detailed structural features, such as the absolute configuration of sugar components, glycosylation position of polyhydroxylated aglycones or interglycosidic linkage in polyglycosides, requires their isolation from the plant material and the application of some additional analytical techniques including nuclear magnetic resonance (NMR) spectroscopy. With the low availability of authentic standards of rare compounds, isolation is also the most reliable way to confirm their pharmacological effects [3,6]. Consequently, combined phytochemical and biological activity studies are necessary to identify and select active markers among numerous candidate constituents of the target plant.

An example of a medicinal plant lacking standardisation procedures is *Gaultheria procumbens* L. (American wintergreen, Ericaceae). This evergreen shrub occurs naturally in northeastern North America and is cultivated worldwide in the temperate zone for ornamental and therapeutic purposes. Leaves, stems, and whole aerial parts of *G. procumbens* are anti-inflammatory, analgesic, and antioxidant agents recommended for treating rheumatoid arthritis, influenza, cold, fever, tracheitis, pharyngitis, pain of various aetiology, and some skin and periodontal diseases [7,8]. The genus *Gaultheria* is known for salicylate glycosides, a rare class of natural analogues of aspirin [7,8,9]. The leading compound, gaultherin (GT), exerts potent anti-inflammatory activity by modulation of pro-inflammatory functions of immune cells, especially secretion of pro-inflammatory cytokines IL-1β, IL-6, and TNF-*α* [10,11]. GT is also known as a cyclooxygenase (COX-2) inhibitor [11], with a potency comparable to synthetic drugs [12]. However, apart from GT, *G. procumbens* accumulates various other polyphenols, which might be co-responsible for the therapeutic effects of the plant. In our previous LC-MS^3^ studies, the leaves and stems of the plant revealed complex matrices with about 50 native polyphenols representing the groups of free catechins, mono- to trimeric procyanidins, flavonol glycosides, and quinic acid pseudodepsides [11,13]. Although there is a solid background of the anti-inflammatory and antioxidant activity of these compounds classes [14,15,16], no data are accessible on the contribution of individual non-salicylate constituents to the pharmacological effects of *G. procumbens*, which is required for the selection of active markers. There is also an information gap on the molecular structures of the *Gaultheria* components. According to our earlier surveys [11,13], at least two primary components of the leaves and stems of *G. procumbens*, a flavonoid diglycoside and a procyanidin A-type trimer, cannot be structurally identified without isolation from the plant. Moreover, in the course of fractionated extraction of the leaves [13], we observed the decreased solubility of the extracts with the increasing presence of the compound identified by LC-MS^3^ with the standard of quercetin 3-glucuronide (miquelianin, MQ), which suggests that the corresponding native constituent might have modified structure. Apart from the structural issues, no fully validated method exists for the quantitative determination of the *G. procumbens* phytochemicals. The first attempt to optimise the appropriate procedure was performed by Saleem et al. [17], who developed an HPLC-PDA-APCI/MSD method to analyse selected polyphenols in various Ericaceae plants, including *G. procumbens*. However, the obtained results are unsatisfactory in terms of peak resolution and peak annotation (some critical peaks co-eluted or were unidentified) and the range of validation tests (only limits of detection and limits of quantitation presented).

Therefore, the present work aimed to select active markers of *G. procumbens* and to develop and fully validate an HPLC-PDA method suitable for QC of the leaf, stem, and aerial parts extracts of the plant. The task was accomplished by applying various chromatographic (analytical and preparative) and spectroscopic techniques and biological capacity tests in an integrated approach. In the first step, 11 model polyphenols were pre-selected based on a UHPLC-PDA-ESI-MS^3^ analysis of the extracts. For complete structural identification, seven of the critical analytes and four minor components were isolated from the plant material by a combination of gel permeation chromatography (GPC), flash chromatography, and preparative HPLC, and analysed by spectroscopic (HR-ESI-MS, ^1^H NMR, ^13^C NMR, COSY, HMQC, HMBC, and ROESY), hydrolytic, thiolytic, and absolute configuration studies. In some cases, additional CD, optical rotation, and flame photometric experiments were performed. Based on the known bioactivity mechanism of *Gaultheria* plants [10,11,12,13,18,19,20,21], the selected compounds were tested in non-cellular and cellular in vitro models for antioxidant and anti-inflammatory activity, including ferric reducing antioxidant power (FRAP), scavenging of superoxide anion (O_2_^•−^), inhibition of pro-inflammatory enzymes (COX-2, and hyaluronidase, HYAL), and modulation of pro-oxidant and pro-inflammatory functions of human neutrophils ex vivo (release of ROS, IL-1β, TNF-α and elastase, ELA-2). The influence of the analytes on the viability of neutrophils was also evaluated. Eventually, the selected analytes were proposed as standardisation markers, and an HPLC-PDA method was developed and validated for quantitative profiling of the extracts.

## 2. Results and Discussion

### 2.1. Selection of Model Compounds and Structural Studies

**Figure 1:**
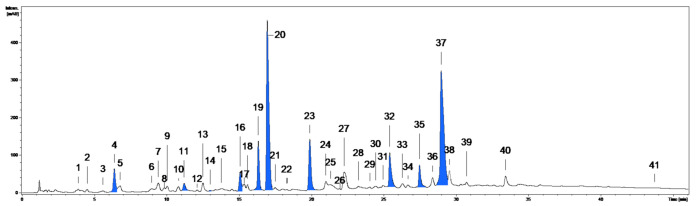
Representative UHPLC-PDA chromatogram of the aerial parts extract ME-AP at 280 nm. The peak numbers refer to those implemented in Table S1. The model compounds (coloured peaks): **4**, NCHA (neochlorogenic acid); **11**, CHA (chlorogenic acid); **14**, CCHA (cryptochlorogenic acid); **16**, PB2 (procyanidin B2); **19**, ECA ((−)-epicatechin); **20**, GT (gaultherin); **23**, CB1 (cinnamtannin B-1); **32**, DGQ (wintergreenoside A); **35**, HY (hyperoside); **37**, MQ (miquelianin); **41**, QU (quercetin).

Among different plant parts of *G. procumbens*, the leaves and aerial parts (whole twigs with leaves) are the most frequently used for medicinal purposes [7,8]. In the present study, the aerial parts were used for QC method development because they combine the chemical matrices of leaves and stems (twigs). The plant material was extracted with methanol-water (75:25, *v*/*v*), the solvent optimised for the recovery of polyphenols from leaves and stems [11,13]. The phytochemical profile of the extract (ME-AP) was studied by UHPLC-PDA-ESI-MS^3^, and the analytes (UHPLC peaks 1–41, Figure 1) were initially identified (Table S1) by comparing their retention times, UV-vis spectra, and MS/MS fragmentation profiles with the standards and literature data [22,23,24]. Considering the previously characterised profiles of leaves and stems [11,13], 11 compounds (coloured peaks in Figure 1) were selected as model structures for *G. procumbens* polyphenols. 

They were assigned as GT, a representative of methyl salicylate glycosides; (−)-epicatechin (ECA) from the group of monomeric flavanols; procyanidin B2 (PB2) and a procyanidin A-type trimer (CB1) as oligomeric proanthocyanidins; chlorogenic acid (CHA), neochlorogenic acid (NCHA), and cryptochlorogenic acid (CCHA), three isomeric caffeoylquinic acids, and MQ, hyperoside (HY), and a quercetin pentoside-glucuronide (DGQ), representing the flavonoid glycosides. Moreover, a flavonoid aglycone quercetin (QU) was added to the set as a useful marker of hydrolytic degradation of the extracts during processing.

Two of the selected compounds, CB1 and DGQ, had to be isolated for complete structural identification. For isolation purposes, the defatted extract (MED-AP) of aerial parts was obtained on a preparative scale and fractionated by liquid-liquid partitioning for basic clean-up [13]. The ballast substances, extracted with chloroform and diethyl ether, were discarded, and the target analytes were concentrated in the fractions of ethyl acetate (EAF-AP), *n*-butanol (BF-AP), and water (WF-AP). Apart from CB1 and DGQ, also some relatively hydrophilic polyphenols, including flavonoid monoglycosides, were present in EAF-AP and BF-AP. However, despite similarities in LC-MS/MS profiles, the solubility of EAF-AP and BF-AP in methanol differed significantly. BF-AP was surprisingly hardly soluble and tended to precipitate some yellowish pigment. To explain this phenomenon, EAF-AP and BF-AP were subjected to GPC to separate the fractions of flavonoids and procyanidins and the sparingly soluble component (MQK) of BF-AP was obtained from the respective flavonoid fraction by crystallisation. Eventually, WF-AP and the procyanidin and flavonoid fractions of EAF-AP were chromatographed using GPC, flash chromatography, and preparative HPLC to yield the target compounds (CB1 and DGQ) and several accompanying constituents (GT, ECA, PB2, MQ, HY, IQ, GV, KG, DGK).

**Figure 2:**
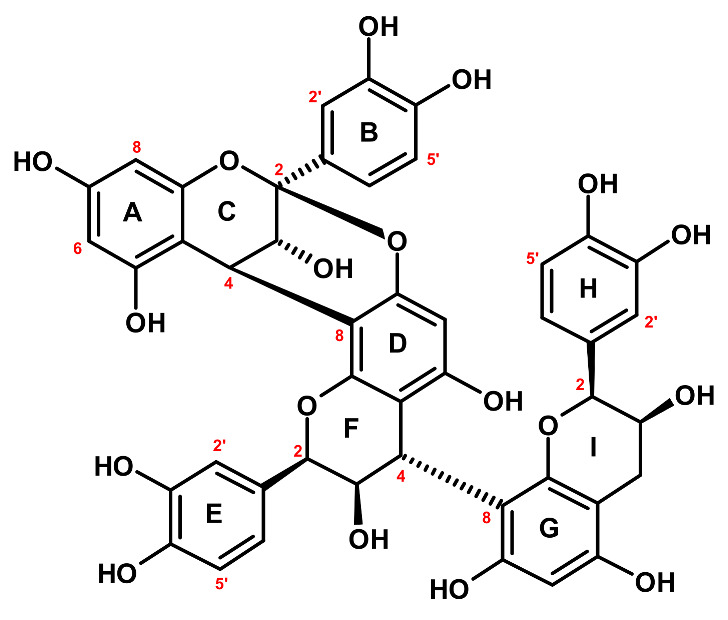
Structure of the isolated procyanidin trimer CB1 (cinnamtannin B-1). Atom numbering in red.

CB1 (Figure 2) showed a UV absorption maximum at 278 nm and a [M − H]^−^ ion at 863 *m*/*z* (LC-PDA-ESI-MS^3^), suggesting that it is an A/B-type proanthocyanidin trimer with one A-type interflavan bond and the molecular formula of C_45_H_36_O_18_ [23]. The secondary ions (negative ions mode) at *m*/*z* 711, 573, 559, 407, 289, and 285 indicated that the trimer is composed of three (epi)catechin units and its fragmentation scheme (Figure S1) was typical of both A- and B-type procyanidins [23,25]. The trimeric structure was confirmed by 45 ^13^C NMR signals (Table S2), including 3 sets of aliphatic carbon signals typical of C-2 (79.9–101.2 ppm), C-3 (68.2–73.6 ppm), and C-4 (29.9–39.3 ppm) positions in the pyran rings [26]. The presence of one A-type interflavan linkage was confirmed by the downfield shift of one of the C-2 signals (101.2 ppm, typical of ketal carbons) in comparison to the other two resonances (79.9 and 81.4 ppm) [26,27]. Moreover, the ^1^H NMR spectrum exhibited the presence of 22 proton resonances (Table S3) against 23 signals required for B-type procyanidins [23,26]. The complete signal assignments of the protons and carbon atoms were achieved using 2D NMR experiments (COSY, HMQC, HMBC). Accordingly, the A-type linkage was confirmed by the presence of two proton doublets at δ_H_ 3.34 ppm (1H, *d*, *J* = 3.4 Hz) and 4.19 ppm (1H, *d*, *J* = 3.4 Hz) corresponding to H-3 and H-4 in the ring C and the lack of H-2. The configuration of the interflavan bonds at C-4(C) and C-4(F) was established as *β* according to the positive Cotton effect in the short wavelength region ([Θ]_228_ + 2.3 × 10^4^) of the CD spectrum, while the negative effect at 270–290 nm ([Θ]_270_ − 2.5 × 10^4^) suggested the *α* orientation of the phenyl rings (B, E, H) [28,29]. One pair of doublets with a small vicinal coupling constant (*J* = 3.4 Hz) corresponding to *trans* relative substitution of H-3(C) and H-4(C), as well as four broad singlets of H-2(F/I) and H-3(F/I), according to *cis* relative substitutions at C-2(C/F/I) and C-3(C/F/I), suggested that CB1 is composed of three (−)-epicatechin subunits [30,31]. The structure of the terminal unit was additionally supported by thiolysis of CB1 and detection of (−)-epicatechin by HPLC-PDA after separation on a chiral column. The specific positions of the B-type linkages between the subunits were established using a combination of 2D NMR through-bond and through-space correlations. The HMBC cross-peaks between the proton H-4(C) and the carbon atoms C-8(D) and C-7(D) indicated the interflavan bond location between the upper and middle subunits. It was further confirmed by the low-temperature ROESY experiment and the correlations between H-2′ and H-6′ of the ring E and H-4 of the ring C, resulting from their spatial proximity and being characteristic of 4→8 linkages [30]. The 4→8 interflavan bond between the middle and the terminal (−)-epicatechin units was established analogously. Eventually, CB1 was identified as (−)-epicatechin-(4*β*→8, 2*β*→*O*→7)-(−)-epicatechin-(4*β*→8)-(−)-epicatechin (cinnamtannin B-1). CB1 is a rare procyanidin that was for the first time isolated from the bark of *Cinnamomum zeylanicum* and is known for its potent antioxidant activity with neuro- and cardioprotective effects [32].

**Figure 3:**
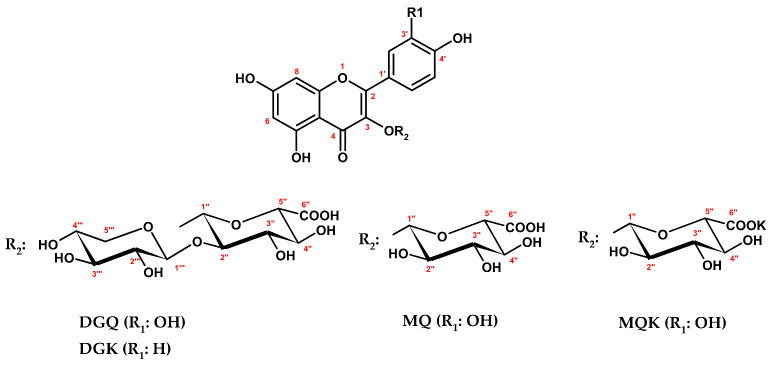
Structures of the isolated flavonoid compounds DGQ (wintergreenoside A), DGK (wintergreenoside B), MQ (miquelianin), and MQK (miquelianin potassium salt). Atom numbering in red.

DGQ (Figure 3) exhibited a UV-vis spectrum with two absorption maxima at 266 nm and 356 nm, diagnostic for flavonol glycosides. The HR-ESI-MS spectrum revealed a [M − H]^−^ ion at 609.1078 *m*/*z*, which agrees with the mass (609.1092) calculated for the molecular formula C_26_H_26_O_17_. The product ions at *m*/*z* 477 [M − 132 − H]^−^, 433 [M − 176 − H]^−^, and 301 [M − 132 − 176 − H]^−^ in LC-PDA-ESI-MS^3^ assay indicated that DGQ is a quercetin pentoside hexuronide. The acid hydrolysis of the glycoside provided an aglycone, identified by HPLC-PDA as quercetin [33], and two carbohydrates identified after conversion to the 1-[(*S*)-*N*-acetyl-methylbenzylamino]-1-deoxy-alditol pentaacetate derivatives [34] as d-xylose and d-glucuronic acid. In the ^1^H NMR spectrum (D_2_O, Table 1), five characteristic aromatic proton signals of quercetin were present [35], as well as two doublets of anomeric protons. One of these protons resonated in a relatively high field (δ_H_ 4.76 ppm), suggesting the presence of a disaccharide moiety and an interglycosidic linkage [35]. Signals in the ^1^H and ^13^C NMR spectra were assigned by 2D experiments (COSY, HMQC, HMBC) and revealed that the terminal sugar is a pentose (d-xylose). The attachment position of the disaccharide at the C-3-OH of the aglycone was concluded from the HMBC cross-peak between the anomeric proton signal of H-1″ (δ_H_ 5.29 ppm) and the carbon resonance of C-3 (δ_C_ 133.5 ppm). The large coupling constants (*J* = 7.5 Hz) observed for both anomeric proton signals, typical of *trans*-diaxial conformation, suggested *β-*configuration of the glycosidic linkage and pyranoside form for both sugar units [35,36]. Two distinctive signals from H-5‴ protons at δ_H_ 3.83 ppm (1H, *dd*, *J*_1_ = 5.7 and *J*_2_ = 10.5 Hz, signal of the equatorial proton H-5_e_‴) and δ_H_ 3.19 ppm (1H, *dd*, *J*_1_ = 10.5 and *J*_2_ = 9.6 Hz, signal of the axial proton H-5_a_‴) further supported the pyranoside form of d-xylose [37]. The ^13^C NMR spectrum (D_2_O, Table 1) showed a sizeable upfield shift (Δδ_C_ = −5.6 ppm) for C-1″ and a downfield shift (Δδ_C_ = +3.6 ppm) for C-2″ when compared to the corresponding resonances of MQ. These shifts located the attachment of d-xylose unit at C-2″ of d-glucuronic acid [36], which was confirmed by the HMBC cross-peak between the anomeric proton signal at δ_H_ 4.77 ppm and a carbon resonance at δ_C_ 80.1 ppm. Eventually, DGQ was identified as quercetin 3-*O*-*β*-d-xylopyranosyl-(1→2)-*β*-d-glucuronopyranoside, a new natural product. The same analysis scheme was applied for DGK (Figure 3, Table 1), a minor component of MED-AP, which was identified as an analogue of DGQ, i.e., kaempferol 3-*O*-*β*-d-xylopyranosyl-(1→2)-*β*-d-glucuronopyranoside, a new natural product as well. The trivial names wintergreenoside A and wintergreenoside B were proposed for DGQ and DGK, respectively.

Compounds MQK and MQ (Figure 3) revealed in LC-PDA-ESI-MS^3^ assay identical UV-vis and mass spectral profiles (Table S1) corresponding to the standard of quercetin 3-glucuronide. On the other hand, both analytes strongly differed in solubility (see above) and melting points (m.p.); in contrast to MQ (m.p. 220–223 °C), MQK showed high m.p. values (>360 °C), not typical of flavonol glycosides. The HPLC-PDA analyses of the acid hydrolysates did not reveal any structural difference between MQ and MQK but confirmed the identity of the aglycone [33] and the carbohydrate unit, including its absolute configuration D [34]. In ^1^H NMR spectra (DMSO-*d*_6_), only the signals expected for quercetin and glucuronic acid were present, however, with a large downfield shift (Δδ_H_ = + 0.76 ppm) for H-2′ and an upfield shift (Δδ_H_ = −0.27 ppm) for H-6′ of MQK in comparison to the corresponding resonances of MQ. In flavonoid glycosides, such phenomenon is usually an effect of structural differences within the sugar moiety [35,36], which suggests that MQK might be an inorganic salt of MQ. Dissociation of the salt in aqueous media, including mobile phases, explains the same retention observed for both compounds in LC. The flame photometric analysis of MQK revealed the presence of potassium ions. It was supported by HR-LSI-MS assay with direct injection of the analyte (in DMSO) to the ion source, which revealed a [M − H]^−^ ion of MQK at *m*/*z* 515.0234, corresponding to the molecular formula C_21_H_17_O_13_K. The remaining structural data on the isolates, such as configuration and position of the glycosidic linkages and pyranose form of the sugar units, were deduced from 1D and 2D NMR (COSY, HMQC, HMBC) experiments. Eventually, MQK and MQ were identified as quercetin 3-*O*-*β*-d-glucuronopyranoside potassium salt and quercetin 3-*O*-*β*-d-glucuronopyranoside, respectively [38,39]. Because of rapid hydrolysis, salts of flavonoid uronides are rarely isolated from plant materials. This is the first report on the occurrence of MQ in the form of potassium salt. Previously, its sodium salt was found in the leaves of *Cyclocarya paliurus* [39].

The spectroscopic (1D and 2D NMR, ESI-MS^3^, UV-vis, CD) and physicochemical studies (optical rotation, absolute configuration of sugar units) on the common extracts components, including GT, ECA, PB2, HY, GV (guaijaverin), IQ (isoquercitrin) and KG (kaempferol 3-*O*-*β*-d-glucuronopyranoside) enabled unequivocal confirmation of their structures [35,37,40,41,42].

### 2.2. Biological Activity of the Selected Gaultheria Polyphenols

The pre-selected compounds were subjected to biological activity tests in vitro to confirm their usefulness as active markers of *G. procumbens* aerial parts. However, among three position isomers of monocaffeoylquinic acid (CHA, NCHA, CCHA), only CHA was tested as a model analyte for the group. The tests were chosen according to the accumulated pharmacological data on *Gaultheria* plants to reflect some of the best recognised mechanisms of their anti-inflammatory and antioxidant activities [10,11,12,13,18,19,20,21]. They included: reducing capacity (FRAP), direct scavenging of O_2_^•−^ as a primary ROS generated by immune cells in inflammatory conditions, direct inhibition of pro-inflammatory enzymes (COX-2, HYAL), and modulation of pro-inflammatory and pro-oxidant functions of human neutrophils ex vivo (influence on the release of ROS, IL-1β, TNF-α, and ELA-2).

**Figure 4:**
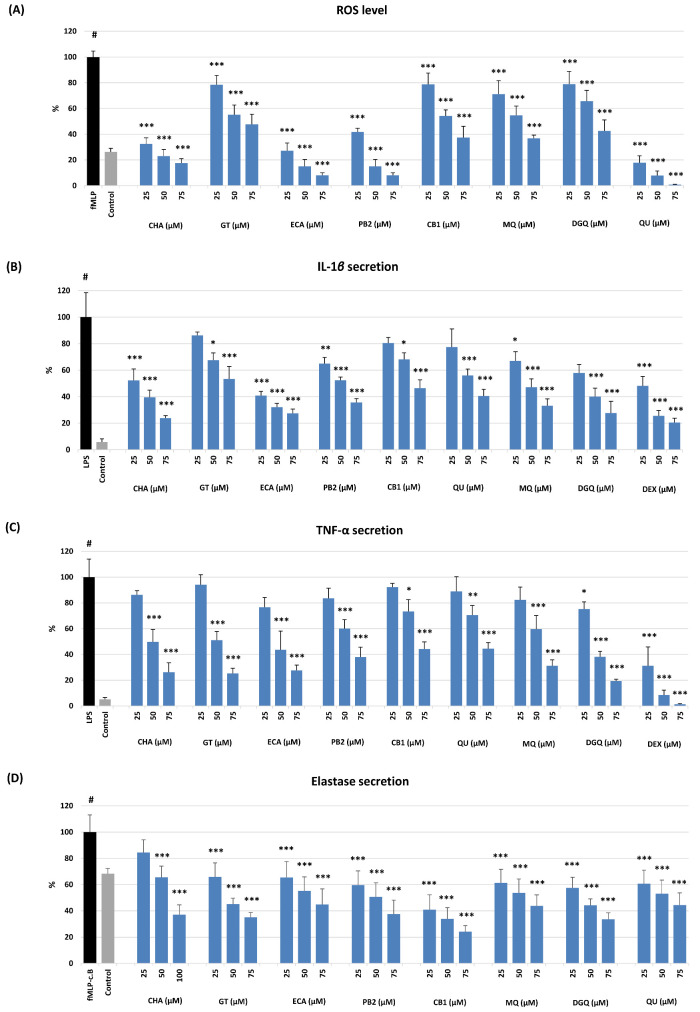
Effect of the selected compounds at 25–75 μM on: (**A**) ROS production and secretion of (**B**) IL-1β, (**C**) TNF-α, and (**D**) ELA-2 by stimulated human neutrophils. Data expressed as means ± SD of three independent experiments performed with cells isolated from five independent donors. Statistical significance: # *p* < 0.001 compared to the non-stimulated control; * *p* < 0.05, ** *p* < 0.01, *** *p* < 0.001 compared to the stimulated control. Analytes: QU, quercetin; MQ, miquelianin; DGQ, wintergreenoside A; ECA, (−)-epicatechin; PB2, procyanidin B2; CB1, cinnamtannin B-1; CHA, chlorogenic acid; GT, gaultherin. Positive controls: (**A**,**D**) QU, quercetin; (**B**,**C**) DEX, dexamethasone.

As shown in Table 2 and Figure 4, the investigated compounds revealed concentration-dependent effects in all models but varied significantly in capacity, depending on the test. The most significant differences were observed in non-cellular models. In comparison to the standards (Trolox, ascorbic acid), all analytes except GT revealed potent antioxidant activity, mostly surpassing that of the standards, with the most substantial effects for all procyanidins (ECA, PB2, CB1) in the O_2_^•−^ scavenging assay, and a flavonol aglycone (QU) and oligomeric procyanidins (PB2, CB1) in the FRAP test (Table 2). The weak capacity observed for GT was not surprising as it lacks free phenolic groups, which are crucial for the direct antioxidant activity of polyphenols [43]. In the case of other analytes, also further structural elements, especially the glycosylation and polymerisation degree, influenced their activity. As the tests were based on different mechanisms (hydrogen atom transfer, HAT, and single electron transfer, SET, respectively), there is no significant correlation between the activity parameters (*r* = 0.5107, *p* > 0.05), which might suggest advantageous complementary antioxidant effects of individual polyphenols in complex oxidant systems.

Similar relationships were observed on the direct inhibition of COX-2, except for relatively lower differences between individual compounds and the potent activity of GT (Table 2). As a derivative of salicylic acid, GT is among the most effective natural COX-2 inhibitors [44]. Indeed, its IC_50_ value fell in between that of two synthetic anti-inflammatory drugs, indomethacin and dexamethasone (Table 2) that might confirm the high potential of GT to treat inflammation. On the other hand, the activity of other tested polyphenols, except for CHA, was only at most twice as weak. Interestingly, the IC_50_ values for COX-2 inhibition were similar for all tested flavonoids and procyanidins. In contrast, the inhibitory activity of the analytes towards HYAL varied in a broader range. Oligomeric procyanidins (PB2, CB1) turned out to be the most potent, and their IC_50_ values did not differ from those of dexamethasone. The HYAL-inhibitory activity of GT was also noticeable and intermediate between the oligomeric procyanidins and all other polyphenols, including flavonoids, ECA, and CHA.

On the other hand, these polyphenols were only up to 1.5-fold less active than GT. As COX-2 and HYAL are involved in the progression of inflammation and therapeutic targets in inflammation-related disorders [45,46], the obtained results might indicate the substantial contribution of all of the assayed compounds to the anti-inflammatory activity of the *Gaultheria* extracts.

As shown in Figure 4, all analytes also revealed antioxidant and anti-inflammatory effects at a cellular level and significantly influenced the pro-oxidant and pro-inflammatory functions of human neutrophils ex vivo with no effect on their viability (Figure S2). Neutrophils are the most abundant immune cells in human blood; in response to infection or tissue injury, they release a large amount of ROS and pro-inflammatory mediators, orchestrating the inflammatory process [47]. All analytes at 25–75 µM inhibited the oxidative burst of *f*MLP-stimulated neutrophils and down-regulated the ROS levels by up to 22–99%, depending on the compound and level (Figure 4A). The most active were QU (a positive control in this test), ECA, CHA, and PB2. Interestingly, the activity of GT did not differ significantly from that of flavonoid glycosides (MQ, DGQ) and a procyanidin trimer (CB1), which confirmed the previous reports on the potent cellular antioxidant effects of salicylates [10,18,21]. The mechanistic studies revealed that these effects are indirect and connected with inhibiting the MAPK/NF-κB pathway [19,21].

Apart from the down-regulation of the oxidative burst, all analytes at the whole concentration range significantly inhibited (*p* < 0.05) the release of IL-1β (Figure 4B) and TNF-α (Figure 4C), two prime agonists of neutrophils and pleiotropic cytokines modulating the gene expression and secretion of numerous other pro-inflammatory factors [48,49], and ELA-2 (Figure 4D), a tissue remodelling enzyme involved in the progression of inflammation [50]. The flavonoid diglycoside (DGQ), ECA, and CHA regulated the levels of IL-1β most substantially by up to 73–76% at 75 µM, but other compounds were also influential. For instance, the weakest activity of GT meant 47% inhibition. Similar effects were revealed in the case of TNF-α release, except that GT was this time among the most active analytes (DGQ, ECA, CHA), exhibiting an inhibition rate up to 75–81% at 75 µM. The relatively smallest differences between the analytes were observed for the ELA-2 release; however, CB1 was clearly the most potent. It down-regulated the enzyme secretion by up to 76% at 75 µM. Nevertheless, the cellular tests indicated that all analytes might be considered active markers of *G. procumbens*, and their contribution to the biological effects of the extracts might depend mainly on their concentration.

### 2.3. Development, Validation, and Application of the HPLC-PDA Method for Quantitative Purposes

**Figure 5:**
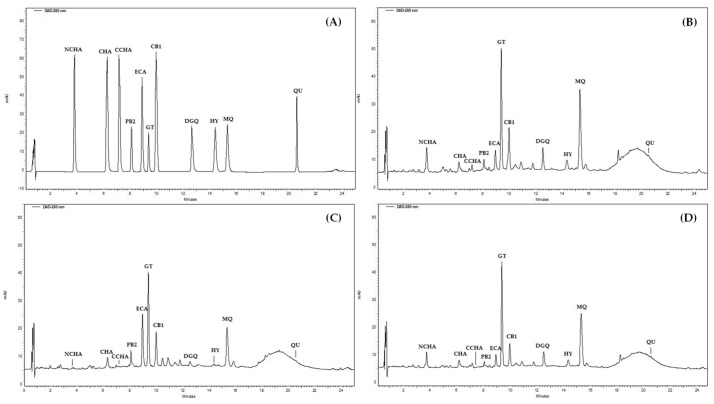
Representative HPLC-PDA chromatograms at 280 nm of: (**A**) standards, and (**B**–**D**) the methanol-water (75:25, *v*/*v*) extracts (ME) of leaves (**B**), stems (**C**), and the aerial parts (**D**) of *G. procumbens* separated under optimised conditions. Analytes: QU, quercetin; MQ, miquelianin; HY, hyperoside; DGQ, wintergreenoside A; ECA, (−)-epicatechin; PB2, procyanidin B2; CB1, cinnamtannin B-1; NCHA, neochlorogenic acid; CHA, chlorogenic acid; CCHA, cryptochlorogenic acid; GT, gaultherin.

The chromatographic procedure was developed to separate the selected 11 constituents of the *G. procumbens* extracts (Figure 5A) in a significantly shorter time than used in the qualitative UHPLC profiling (80 min including equilibration). An RP-18 fused-core column was chosen for the task due to its high performance in HPLC systems [51]. Eventually, a linear gradient of acetonitrile in acidified water (Table 3), column temperature (18 °C), and flow rate (1.4 mL/min) were optimised to achieve satisfactory separation of the analytes within 35 min (including equilibration).

The proposed method was validated by determining the selectivity, linearity, precision, and accuracy according to the International Council for Harmonization (ICH) Guidance for Industry [52]. The adequate selectivity was demonstrated in the real samples (Figure 5B–D): the target peaks eluted as pure bands and were satisfactorily separated from the matrix. The linearity was confirmed in the whole concentration range, including limits of quantitation (LOQs), with *r* > 0.9994 (Table 4). The statistical significance of the regression equations was verified in the *F*-test (*p* < 0.05). The low limits of detection (LODs, 0.11–0.55 µg/mL, 0.53–2.76 ng) and LOQs (0.36–1.84 µg/mL, 1.79–9.19 ng) demonstrated the high sensitivity of the method (Table 4). The RSD values measured for peak area for intra-day (0.39–1.93%) and inter-day precision (2.74–4.12%) did not exceed the predicted values (2.19–4.38%), calculated using the Horvitz equation according to the AOAC International [53], which indicated the adequate precision (Table 5). In the accuracy studies, the recoveries were within the range of 95.45–101.16% (Table 5), thus within the acceptance limits (92–105%) [54]. Finally, the proposed method is superior to the previously published approach [17] in terms of the range of the tested analytes, chromatographic performance, and validation parameters.

The applicability of the method to the intended use was demonstrated by determining the levels of the target compounds in the aerial part, leaf, and stem extracts of *G. procumbens* (Table S2). The crude extracts and the concentrated fractions obtained for preparative purposes were analysed to cover a wide range of concentrations and relative ratios between the analytes.

Their total content in the crude extracts varied in a narrow range of 181.04–192.78 mg/g, but it rose to 384.48 mg/g in the fractions (EAF-AP), confirming that the selected model compounds form a large part of the extracts.

Moreover, the increase in the QU content from 0.19 mg/g dw in ME-AP to 0.48 mg/g dw in MED-AP and 5.36–8.68 mg/g dw in its organic fractions confirmed that QU might indeed be a suitable marker of the hydrolytic degradation of the extracts during processing or storage. Considering the demonstrated activity of the selected compounds, they might be recommended as standardisation markers for the quality control of the analysed extracts.

## 3. Materials and Methods

### 3.1. Reagents and Standards

HPLC-grade reagents and standards, such as quercetin dihydrate (QU); hyperoside (HY; quercetin 3-*O*-*β*-d-galactopyranoside); miquelianin (MQ; quercetin 3-*O*-*β*-d-glucuronopyranoside); guaijaverin (GV; quercetin 3-*O*-*α*-l-arabinopyranoside); isoquercitrin (IQ; quercetin 3-*O*-*β*-d-glucopyranoside); (−)-epicatechin (ECA); kaempferol 3-*O*-*β*-d-glucuronopyranoside (KG); procyanidin B2 (PB2; epicatechin-(4*β*→8)-epicatechin); 3-*O*-caffeoyl-quinic acid (NCHA, neochlorogenic acid); 4-*O*-caffeoylquinic acid (CCHA, cryptochlorogenic acid); 5-*O*-caffeoylquinic acid hemihydrate (CHA, chlorogenic acid); bovine testis hyaluronidase; bovine serum albumin; hyaluronic acid; ascorbic acid (AA); dexamethasone (DEX); indomethacin (IND); (±)-6-hydroxy-2,2,7,8-tetramethylchroman-2-carboxylic acid (Trolox^®^, TX); *N*-formyl-l-methionyl-l-leucyl-l-phenylalanine (*f*MLP); lipopolysaccharide (LPS) from *Escherichia coli*; *N*-succinyl-alanine-alanine-valine *p*-nitroanilide (SAAVNA); formic acid; acetic acid, and orthophosphoric acid were of HPLC-grade and purchased from Merck (Darmstadt, Germany). HPLC-grade solvents (acetonitrile, methanol, hexane, isopropanol, and water) were from Avantor Performance Materials (Gliwice, Poland). Solvents used to extract the plant material and purify the compounds were from Chempur (Piekary Śląskie, Poland) and were of analytical grade. 

### 3.2. Plant Material and Extraction

Leaves, stems, and aerial parts of *G. procumbens* L. were collected in October 2019 in the gardening centre of Ericaceae plants, Gospodarstwo Szkolkarskie Jan Cieplucha (54°44′ N, 19°18′ E), Konstantynow Lodzki (Poland), where the plants grew in an open area. The plant origin and authentication were described previously [11]. The voucher specimens (KFG/HB/19001-GPRO-LEAF, KFG/HB/19001-GPRO-STEM, KFG/HB/19001-GPRO-AP) were deposited in the Medicinal Plant Garden, Medical University of Lodz (Poland). Samples of the plant material were air-dried at 35 °C in the shade with air humidity of about 40%, powdered with an electric grinder, and sieved through a ø 0.315 mm sieve.

The powdered plant material (100 g each) was subjected to static solvent extraction and refluxed thrice (2 h each time) with 300 mL of methanol-water (75:25, *v*/*v*). Then, the combined extracts were evaporated at 40 °C (*in vacuo*) and next lyophilised (Alpha 1-2/LD Plus freeze dryer, Christ, Osterode am Harz, Germany) to provide the methanol-water extracts (ME) of the leaves (ME-L, 37.2 g dw), stems (ME-S, 45.7 g dw), and aerial parts (ME-AP, 42.3 g dw), respectively.

For isolation of polyphenols, the sample of the aerial parts (2730 g) was pre-extracted with chloroform in Soxhlet apparatus (1 L, 48 h) to remove lipoidal components and refluxed exhaustively with methanol-water (75:25, *v*/*v*) to obtain the defatted methanol-water extract (MED-AP, 743.8 g dw). Eventually, MED-AP was fractionated into the diethyl ether fraction (DEF-AP, 25.3 g dw), ethyl acetate fraction (EAF-AP, 30.5 g dw), *n*-butanol fraction (BF-AP, 173.1 g dw), and water fraction (WF-AP, 419.9 g dw) according to Michel et al. [13]. The obtained extract and fractions, except WF-AP, were evaporated *in vacuo*, and the water-containing ones (MED-AP, BF-AP, WF-AP) were next lyophilised. The extraction yields were calculated per dry weight (dw) of the plant material.

### 3.3. UHPLC-PDA-ESI-MS^3^ Profiling

The profile of ME-AP was analysed by UHPLC-PDA-ESI-MS^3^ according to Michel et al. [13] using the same equipment and chromatographic procedure. Before the analysis, samples of the tested extracts (10–40 mg) were dissolved in 10 mL of methanol-water (75:25, *v*/*v*) and filtered through a PTFE syringe filter (25 mm, 0.2 µm, Ahlstrom, Helsinki, Finland).

### 3.4. Isolation of Phenolic Compounds

#### 3.4.1. Preparative HPLC-PDA

The experiments were performed on preparative Waters 2545 binary system (Milford, MA, USA) equipped with an autosampler, a Waters 2998 PDA detector, and a preparative Zorbax SB C18 column (5.0 µm, 150 mm × 21.2 mm; Agilent Technologies, Santa Clara, CA, USA). The mobile phase consisted of solvent A (water-formic acid, 100:0.1, *v*/*v*) and solvent B (acetonitrile-formic acid, 100:0.1, *v*/*v*). The elution profile was as follows: 0–30 min, 5–25.5% B (*v*/*v*); 30–35 min, 25.5–5% B (equilibration). All gradients were linear. The separation was carried out at room temperature (20–25 °C), the flow rate was 7 mL/min, and the injection volume was 250 µL. Before the isolation, the phenolic fractions (portions of 50 mg) were dissolved in the mobile phase (1 mL) and filtered through a PTFE syringe filter (25 mm, 0.2 µm, Ahlstrom, Helsinki, Finland). The fraction collection was triggered automatically by the UV signal at λ = 280 nm (for proanthocyanidin and GT fractions) or 350 nm (for flavonoid fractions). The separation was repeated several times for each fraction, and the eluates containing the respective analytes were combined.

#### 3.4.2. Flash Chromatography

The experiments were carried out using a VersaFlash HTFP system equipped with a high flow VersaFlash Piston Pump and a VersaPak^TM^ C18 Cartridge (150 mm × 40 mm) (Supelco, Merck, Darmstadt, Germany). The mobile phase consisted of solvent A (methanol) and solvent B (water-formic acid, 100:0.1, *v*/*v*). The elution profile was as follows: 0–60 min, 20–40% A (*v*/*v*, stepwise gradient, concentration changed by 2.5% every 7.5 min.). Before the isolation, the analysed fractions (portions of 500 mg) were dissolved in 3 mL of methanol-water (2:8, *v*/*v*) and filtered through a PTFE syringe filter (25 mm, 0.2 µm, Ahlstrom, Helsinki, Finland). The separation was carried out at room temperature (20–25 °C) and a 20 mL/min flow rate. The process was repeated several times, and the eluates presenting similar HPLC-PDA profiles (Section 3.7) were combined.

#### 3.4.3. Isolation Procedure

The sample of EAF-AP (10.4 g) was dissolved in a small amount of methanol and separated by GPC on Sephadex LH-20 (100 g; 80 cm × 3 cm; Merck, Darmstadt, Germany) using methanol for elution. Based on the chromatographic profile (HPLC-PDA, Section 3.7), eluates were combined to provide several proanthocyanidin and flavonoid fractions, which were independently subjected to preparative HPLC separation (Section 3.4.1) to obtain the pure compounds ECA (152 mg), PB2 (27.4 mg), and CB1 (200 mg) from the proanthocyanidin fractions and HY (175 mg), MQ (141 mg), and GV (37 mg) from the flavonoid fractions. The isolated proanthocyanidins were lyophilised, and the flavonoids were crystallised from methanol or methanol-water (7:3, *v*/*v*).

The sample of BF-AP (4.7 g) was chromatographed on Sephadex LH-20 (as described for EAF-AP) to separate the flavonoid fraction, which after crystallisation from methanol afforded compound MQK (125 mg).

The WF-AP (5.7 g) sample was separated using flash chromatography (Section 3.4.2) to isolate the fractions of salicylates and flavonoid diglycosides. The fractions were purified on Sephadex LH-20 (30 g; 40 × 2 cm), using methanol for elution, to provide compounds GT (125.2 mg), DGQ (38.2 mg), and DGK (10.1 mg), respectively.

### 3.5. Structure Elucidation

Melting points (uncorrected) were determined on a Boetius apparatus (Carl Zeiss, Jena, Germany). The UV-vis spectra were recorded in methanol at 25 °C on a UV-1601 spectrophotometer (Rayleigh, Beijing, China). The optical rotation (${\left[\alpha \right]}_{D}^{20}$) was measured in methanol on a PolAAr 3001 polarimeter (Optical Activity, Ramsey, U.K.). The CD spectra were recorded on a Jobin-Yvon CD6 spectrometer (Horiba Scientific, Edison, NJ, USA). The analysis of metal ions was performed on a 410 Flame photometer (Sherwood Scientific, Cambridge, UK).

The ^1^H NMR, ^13^C NMR, ^1^H-^1^H COSY, HMQC, and HMBC spectra were recorded at 25 °C on a Bruker Daltonik III 600 spectrometer (Bruker BioSpin Co., Billerica, MA, USA) in methanol-*d*_4_, DMSO-*d*_6_, or water-*d*_2_ (600 MHz for ^1^H and 150.9 MHz for ^13^C), with TMS as the internal standard. The ROESY spectrum was recorded at −30 °C in acetone-*d*_6_. The HR-MS spectra were recorded on an AutoSpec Premier Mass Spectrometer (Waters, Milford, MA, USA) coupled with an HP 7890 gas chromatograph (Agilent, Santa Clara, CA, USA).

The acid hydrolysis of glycosides and identification of the absolute configuration of free monosaccharides after their conversion to 1-[(*S*)-*N*-acetyl-α-methylbenzylamino]-1-deoxy-alditol pentaacetate derivatives was performed as described earlier [34].

The process of epimerisation was performed according to Seto et al. [55], with slight modifications. First, the sample of (+)-catechin (50 mg) was dissolved in 5 mL of a McIlvaine buffer (pH = 5.0, containing 0.2 mol/L disodium phosphate and 0.1 mol/L citric acid, 1:1, *v*/*v*) and then autoclaved for 30 min at 120 °C. After cooling to room temperature, the reaction mixture was evaporated to dryness *in vacuo* and dissolved in methanol (1 mL). The product formed from (+)-catechin, i.e., (+)-epicatechin (15 mg), was isolated by GPC on Sephadex LH-20 (60 g; 80 cm × 3 cm) with methanol as an eluent and finally purified by crystallisation from methanol. The structure was confirmed by NMR studies and optical rotation analysis [55].

The thiolysis was performed according to Meagher et al. [56]. Briefly, 200 µL of the solution of hydrochloric acid in methanol (3.3:96.7, *v*/*v*) and 400 µL of the solution of benzyl mercaptan in methanol (5:95, *v*/*v*) was added to the vials containing 200 µL of the solutions of CB1 and PB2 in methanol (4 mg/mL each). The mixtures were heated at 40 °C for 30 min in a heating block and cooled to room temperature. The obtained thiolysates were immediately analysed by HPLC-PDA versus the standards of (−)-epicatechin and (+)-epicatechin, obtained in the epimerisation process. The analyses were performed at 25 °C on the Hitachi HPLC system (Section 3.7) using a chiral column Lux Cellulose-2 (5.0 μm, 150 mm × 4.6 mm; Phenomenex, Torrance, CA, USA). The mobile phase was hexane-isopropanol-50% (*w*/*w*) acetic acid (40:60:1, *v*/*v*/*v*, isocratic elution). The flow rate was 0.6 mL/min, the injection volume was 20 µL, and detection was set at 280 nm.

Compound MQ; quercetin 3-*O*-*β*-d-glucuronopyranoside (miquelianin). Yellow needles: m.p. 220–223 °C; UV (methanol) λ_max_ nm: 256, 298, 358; ESI-MS^2^ *m*/*z* (intensity): [M + H]^+^ 479 (100), MS^2^: [M + H-glucuronic acid]^+^ 303 (64.1); [M − H]^−^ 477 (100), MS^2^: [M − H-glucuronic acid]^−^ 301 (91.5); ^1^H NMR (methanol-*d*_4_) δ ppm: 7.75 (1H, *d*, *J* = 2.3 Hz, H-2′); 7.64 (1H, *dd*, *J*_1_ = 2.3 Hz, *J*_2_ = 8.3 Hz, H-6′); 6.89 (1H, *d*, *J* = 8.3 Hz, H-5′); 6.44 (1H, *d*, *J* = 2.3 Hz, H-8); 6.25 (1H, *d*, *J* = 2.3 Hz, H-6); 5.38 (1H, *d*, *J* = 7.5 Hz, H-1″); 3.76 (1H, *d*, *J* = 9.8 Hz, H-5″); 3.63 (1H, *dd*, *J*_1_ = 9.0 Hz, *J*_2_ = 9.8 Hz, H-4″); 3.56 (1H, *dd*, *J*_1_ = 7.5 Hz, *J*_2_ = 9.0 Hz, H-2″); 3.52 (1H, *dd*, *J*_1_ = 9.0 Hz, *J*_2_ = 9.0 Hz, H-3″); ^13^C NMR (methanol-*d*_4_) δ ppm: 160.1 (C-2); 136.6 (C-3); 180.3 (C-4); 164.0 (C-5); 100.9 (C-6); 167.0 (C-7); 95.8 (C-8); 159.5 (C-9); 106.7 (C-10); 123.9 (C-1′); 118.5 (C-2′); 146.9 (C-3′); 150.9 (C-4′); 117.1 (C-5′); 124.4 (C-6′); 105.4 (C-1″); 76.5 (C-2″); 78.8 (C-3″); 73.9 (C-4″); 78.2 (C-5″); 174.1 (C-6″); ^1^H NMR (DMSO-*d*_6_) δ ppm: 7.70 (1H, *d*, *J* = 2.3 Hz, H-2′); 7.56 (1H, *dd*, *J*_1_ = 2.3 Hz, *J*_2_ = 8.3 Hz, H-6′); 6.85 (1H, *d*, *J* = 8.3 Hz, H-5′); 6.42 (1H, *d*, *J* = 2.3 Hz, H-8); 6.22 (1H, *d*, *J* = 2.3 Hz, H-6); 5.46 (1H, *d*, *J* = 7.5 Hz, H-1″); 3.56 (1H, *d*, *J* = 9.8 Hz, H-5″); 3.37 (1H, *dd*, *J*_1_ = 8.7 Hz, *J*_2_ = 9.8 Hz, H-4″); 3.25–3.33 (2H, *m*, H-2″ and H-3″). For trivial atom numbering see chemical formula of MQ (Figure 3).

Compound MQK; quercetin 3-*O*-*β*-d-glucuronopyranoside potassium salt (miquelianin potassium salt). Pale yellow needles: m.p. > 360 °C; UV (75% *v*/*v* aqueous methanol) λ_max_ nm: 256, 299, 361; HR-ESI-MS *m*/*z*: [M − H]^−^ 515.0234 (C_21_H_17_O_13_K); ESI-MS^2^ *m*/*z* (intensity): [M + H]^+^ 479 (100), MS^2^: [M + H-glucuronic acid]^+^ 303 (64.1); [M − H]^−^ 477 (100), MS^2^: [M − H-glucuronic acid]^−^ 301 (91.5); flame photometry (K^+^ ions); ^1^H NMR (DMSO-*d*_6_) δ ppm: 8.46 (1H, *d*, *J* = 2.3 Hz, H-2′); 7.29 (1H, *dd*, *J*_1_ = 2.3 Hz, *J*_2_ = 8.3 Hz, H-6′); 6.82 (1H, *d*, *J* = 8.3 Hz, H-5′); 6.35 (1H, *d*, *J* = 2.3 Hz, H-8); 6.17 (1H, *d*, *J* = 2.3 Hz, H-6); 5.23 (1H, *d*, *J* = 7.5 Hz, H-1″); 3.39 (1H, *d*, *J* = 9.8 Hz, H-5″); 3.27 (1H, *dd*, *J*_1_ = 8.7 Hz, *J*_2_ = 9.8 Hz, H-4″); 3.25 (1H, *dd*, *J*_1_ = 7.5 Hz, *J*_2_ = 8.7 Hz, H-2″); 3.21 (1H, *dd*, *J*_1_ = 8.7 Hz, *J*_2_ = 8.7 Hz, H-3″); ^13^C NMR (DMSO-*d*_6_) δ ppm: 157.9 (C-2); 134.2 (C-3); 177.7 (C-4); 161.1 (C-5); 99.2 (C-6); 165.2 (C-7); 93.9 (C-8); 156.7 (C-9); 103.7 (C-10); 120.6 (C-1′); 118.6 (C-2′); 144.9 (C-3′); 148.5 (C-4′); 115.6 (C-5′); 120.4 (C-6′); 103.5 (C-1″); 74.0 (C-2″); 76.9 (C-3″); 71.9 (C-4″); 74.5 (C-5″); 172.2 (C-6″). For trivial atom numbering see chemical formula of MQK (Figure 3).

Compound DGQ; quercetin 3-*O*-*β*-d-xylopyranosyl-(1→2)-*β*-d-glucuronopyranoside (wintergreenoside A). Yellow amorphous solid: m.p. 217–221 °C; UV (methanol) λ_max_ nm: 266, 282, 356; HR-ESI-MS *m*/*z*: [M − H]^−^ 609.1078 (C_26_H_25_O_17_); ESI-MS^2^ *m*/*z* (intensity): [M + H]^+^ 611 (100), MS^2^: [M + H-xylose]^+^ 479 (53.9), [M + H-xylose-glucuronic acid]^+^ 303 (42.1); [M − H]^−^ 609 (100), MS^2^: [M − H-xylose-glucuronic acid]^−^ 301 (68.4). ^1^H and ^13^C NMR data (methanol-*d*_4_ and water-*d*_2_): see Table 1.

Compound DGK; kaempferol 3-*O*-*β*-d-xylopyranosyl-(1→2)-*β*-d-glucuronopyrano-side (wintergreenoside B). Yellow amorphous solid: m.p. 210–217 °C; UV (methanol) λ_max_ nm: 260, 300, 344; HR-ESI-MS *m*/*z*: [M − H]^−^ 593.1136 (C_26_H_25_O_16_); ESI-MS^2^ *m*/*z* (intensity): [M + H]^+^ 595 (100), MS^2^: [M + H-xylose]^+^ 463 (72.2), [M + H-xylose-glucuronic acid]^+^ 287 (51.9); [M − H]^−^ 593 (100), MS^2^: [M − H-xylose-glucuronic acid]^−^ 285 (82.1). ^1^H and ^13^C NMR data (methanol-*d*_4_): see Table 1.

Compound CB1; (−)-epicatechin-(4*β*→8, 2*β*→*O*→7)-(−)-epicatechin-(4*β*→8)-(−)-epi-catechin (cinnamtannin B-1). Pale pink amorphous solid: m.p. 202–204 °C; ${\left[\alpha \right]}_{D}^{20}$ = +93.0° (*c* = 1.00 g/100 mL, methanol); UV (methanol) λ_max_ nm: 278; CD (*c* = 1.57 mmol/L, methanol) [Θ]_199_ +140275, [Θ]_207_ −380803, [Θ]_228_ +23491, [Θ]_239_ +165245sh, [Θ]_270_ −25296; ESI-MS^2^ *m*/*z* (intensity): [M + H]^+^ 865 (100), MS^2^: [M + H − 152]^+^ 713 (57.6); [M − H]^−^ 863 (100), MS^2^: [M − H − 152]^−^ 711 (63.5). ^1^H and ^13^C NMR data (methanol-*d*_4_): see Table S3.

### 3.6. Biological Activity Tests

#### 3.6.1. Non-Cellular In Vitro Models

The FRAP was determined according to Olszewska and Michel [57] and expressed in mol of ferrous ions (Fe^2+^) produced by 1 mol of an analyte, calculated from the calibration curve of ferrous sulphate. The O_2_^•−^ scavenging capacity was evaluated according to Michel et al. [13] and expressed as SC_50_ values, calculated from the concentration-scavenging curve. The ability of the analytes to inhibit COX-2 and HYAL was evaluated by ELISA test following the manufacturer’s instructions (Cayman Chemical, Ann Arbor, MI, USA) and according to Matczak et al. [58], respectively, and expressed as IC_50_ values, calculated from the concentration-inhibition curves. The analytes were tested at the final concentrations of 1.5–65 µM, 1.5–1500.0 µM, 0.15–3.70 mM, and 5.0–200 µM for the FRAP, O_2_^•−^, COX-2, and HYAL inhibition assays, respectively. The standards of ascorbic acid and Trolox (FRAP, O_2_^•−^ scavenging) or dexamethasone and indomethacin were used as positive controls in antioxidant and anti-inflammatory activity tests, respectively. A SPECTROstar Nano (BMG Labtech GmbH, Ortenberg, Germany) microplate reader and 96-well plates were used for the measurements.

#### 3.6.2. Cellular Model of Human Neutrophils Ex Vivo

Neutrophils were isolated from buffy coat fractions of human blood purchased from the Warsaw Blood Donation Centre. The blood samples were collected from healthy adult human donors (18–35 years old), and routine laboratory tests showed all values within the normal ranges. The study conformed to the principles of the Declaration of Helsinki (the approval of the bioethics committee is not required).

The isolation was performed using the dextran sedimentation method before hypotonic lysis of erythrocytes and centrifugation in a Ficoll Hypaque gradient as previously described [11]. The purity of the cells exceeded 97%.

The potential cytotoxicity (influence on cell wall integrity) of the analytes was evaluated by flow cytometry using propidium iodide (PI) staining according to Michel et al. [11]. The analytes were tested at the levels of 25–75 µM. The results were expressed as a percentage of PI(+) cells (displaying high permeability to PI).

The influence of the analytes on the pro-oxidant and pro-inflammatory functions of neutrophils was evaluated as described previously [11,18]. Briefly, the ROS level in *f*MLP-stimulated neutrophils was determined by the luminol-dependent chemiluminescence method; the ELISA tests measured the release of IL-1β and TNF-α from LPS-stimulated neutrophils following the manufacturer’s instruction (BD Biosciences, San Jose, CA, USA), and the secretion of ELA-2 from neutrophils stimulated by *f*MLP and cytochalasin B was determined using SAAVNA as a substrate. The standards of QU (ROS, ELA-2) or dexamethasone (IL-1β, TNF-α) were used as positive controls, respectively. The analytes were tested at the levels of 25–75 µM. The assays were performed using 96-well plates and a microplate reader (Synergy 4, BioTek, Winooski, VT, USA).

### 3.7. Quantitative HPLC-PDA Assay

The analyses were performed on an HPLC VWR-Hitachi LaChrom Elite^®^ System equipped with a PDA Detector (scanning in the wavelength range of 220–450 nm), thermostated autosampler, and column compartments, and a quaternary gradient pump (VWR, Hitachi, Tokyo, Japan). Separations were carried out on a C18 Ascentis^®^ Express column (2.7 μm, 75 mm × 4.6 mm i.d.; Supelco, Merck, Darmstadt, Germany), guarded by a C18 Ascentis^®^ C18 Supelguard guard column (3 μm, 20 mm × 4 mm i.d.; Supelco). The elution system consisted of solvent A (water-85% orthophosphoric acid, 100:0.5, *v*/*w*, pH 2.0) and solvent B (acetonitrile). The optimised elution profile is shown in Table 3. The flow rate was 1.4 mL/min, the injection volume was 5 µL, and the column was maintained at 18 °C. The detection wavelength was set at 280, 285, 325, 350, or 370 nm, depending on the analyte (Table 4). Before injection, samples of the extracts (1–50 mg) were dissolved in 10 mL of methanol-water (75:25, *v*/*v*) and filtered through a PTFE syringe filter (25 mm, 0.2 µm, Ahlstrom, Helsinki, Finland). Results were calculated in mg/g dw of the extracts.

### 3.8. Validation of the HPLC-PDA Method

The analytical method was validated by determining the selectivity, linearity, precision, accuracy, and stability of each analyte [52].

The method’s selectivity and peak purity were evaluated by comparison of the retention times and UV-vis spectra with reference standards using an automated match system.

Linearity was tested using the standard stock solution containing eleven reference compounds, dissolved and serially diluted (in triplicate) with methanol-water (75:25, *v*/*v*) to six concentration levels (2%, 10%, 25%, 50%, 75%, and 100% of the stock concentration). Each replicate solution was injected into the HPLC system in triplicate. Two linear regression models were tested (y = *a*x + *b*; y = *a*x), and the *F*- and *t*-tests were applied to check the statistical significance of the regression equations, slopes, and intercepts at a 99% confidence level.

The LOD and LOQ values were determined using a serial dilution of the standard solution. The lowest concentrations with the signal-to-noise ratio (S/N) of 3 were accepted as LODs, while the levels with S/N above 10 were accepted as LOQs if the RSD values for peak area were not higher than 15%.

The repeatability (intra-day variability) and the intermediate precision (inter-day variability) were tested for retention times and peak areas using the standard solution at 10%, and 100% of the stock concentration and the aerial parts extract of *G. procumbens* (ME-AP). The repeatability was determined by triplicate analysis of each sample within 24 h, while the intermediate precision was evaluated on three non-consecutive days within two weeks.

The accuracy was tested in the real sample ME-AP by the standard addition/recovery procedure at three different levels of each standard, within the analytical range investigated. The samples were prepared in triplicate by spiking the extract with the standard solution. The replicate samples were analysed in triplicate. The accuracy was calculated as the mean recovery of the analytes from the spiked versus the non-spiked extracts.

### 3.9. Statistical Analysis

The results were expressed as means ± standard deviation (SD) for replicate determinations. The statistical analyses (calculation of SD, one-way analysis of variance, HSD Tukey tests, and linearity studies) were performed using the Statistica12Pl software for Windows (StatSoft Inc., Krakow, Poland), with *p* values less than 0.05 being regarded as significant.

## 4. Conclusions

This work is the first integrated phytochemical and biological activity study of individual polyphenols of *G. procumbens* aerial parts, leaf, and stem extracts. The LC-MS/MS analysis, isolation, and spectroscopic experiments led to the selection and complete structural identification of eleven model compounds representing all classes of *Gaultheria* polyphenols, including one new natural product. In addition, the second new natural compound was identified among minor components of the plant. The biological activity tests revealed that the model constituents significantly down-regulated the pro-oxidant and pro-inflammatory functions of human neutrophils, inhibited two key pro-inflammatory enzymes, and most of them, except GT, exerted potent direct antioxidant capacity. As these mechanisms have been previously connected to the health benefits of *Gaultheria* plants [10,11,12,13,18,19,20,21], all eleven compounds might be considered active markers for the standardisation of the respective extracts. Therefore, the proposed HPLC-PDA method, fully validated during the study using these markers as calibration standards, might be recommended as a simple, accurate and reproducible tool for that purpose.

## Figures and Tables

**Table 1:** NMR spectral data of compound DGQ in methanol-*d*_4_ and water-*d*_2_ and compound DGK in methanol-*d*_4_ (600 MHz for ^1^H and 150.9 MHz for ^13^C) ^a^.

DGQ					DGK		
Pos. ^b^	δ_H_ ^c^	δ_C_ ^c^	δ_H_ ^d^	δ_C_ ^d^	Pos. ^b^	δ_H_ ^c^	δ_C_ ^c^
2		157.3		156.8	2		157.2
3		133.8		133.5	3		133.5
4		178.1		177.4	4		178.1
5		161.5		159.7	5		161.7
6	6.17 (1H, *d*, *J* = 1.9)	98.5	5.89 (1H, *d*, *J* = 1.7)	98.7	6	6.21 (1H, *d*, *J* = 1.9)	98.4
7		164.4		162.6	7		164.4
8	6.36 (1H, *d*, *J* = 1.9)	93.3	5.95 (1H, *d*, *J* = 1.7)	94.3	8	6.41 (1H, *d*, *J* = 1.9)	93.3
9		156.9		155.9	9		157.2
10		104.4		104.3	10		104.5
1′		121.7		121.5	1′		121.4
2′	7.66 (1H, *d*, *J* = 1.9)	116.2	7.27 (1H, *d*, *J* = 1.7)	116.4	2′, 6′	8.08 (2H, *d*, *J* = 9.0)	130.9
3′		144.6		143.4	3′, 5′	6.91 (2H, *d*, *J* = 9.0)	114.8
4′		148.4		147.3	4′		160.1
5′	6.88 (1H, *d*, *J* = 8.3)	114.8	6.65 (1H, *d*, *J* = 8.9)	115.3			
6′	7.59 (1H, *dd*, *J*_1_ = 1.9, *J*_2_ = 8.3)	122.1	7.15 (1H, *dd*, *J*_1_ = 1.7, *J*_2_ = 8.9)	122.6			
	3-*β*-d-glucuronopyranosyl:						
1″	5.54 (1H, *d*, *J* = 7.5)	99.8	5.29 (1H, *d*, *J* = 7.5)	99.5	1″	5.58 (1H, *d*, *J* = 7.2)	99.6
2″	3.80 (1H, *dd*, *J* = 7.5, *J*_2_ = 9.0)	80.1	3.82 (1H, *dd*, *J* = 7.5, *J*_2_ = 8.3)	79.4	2″	3.74 (1H, *dd*, *J* = 7.2, *J*_2_ = 9.4)	80.5
3″	3.66 (1H, *dd*, *J*_1_ = 9.0, *J*_2_ = 9.8)	71.6	3.64 (1H, *dd*, *J*_1_ = 8.3, *J*_2_ = 9.0)	71.5	3″	3.63 (1H, *dd*, *J*_1_ = 9.4, *J*_2_ = 9.8)	71.5
4″	3.68 (1H, *dd*, *J*_1_ = 9.8, *J*_2_ = 10.2)	76.7	3.70 (1H, *dd*, *J*_1_ = 9.0, *J*_2_ = 9.4)	76.4	4″	3.67 (1H, *dd*, *J*_1_ = 9.8, *J*_2_ = 10.2)	76.4
5″	3.74 (1H, *d*, *J* = 10.2)	75.4	3.80 (1H, *d*, *J* = 9.4)	76.0	5″	3.71 (1H, *d*, *J* = 10.2)	75.6
6″		173.4		174.5	6″		173.7
	2″-*β*-d-xylopyranosyl:						
1‴	4.77 (1H, *d*, *J* = 7.5)	103.8	4.76 (1H, *d*, *J* = 7.5)	103.1	1‴	4.78 (1H, *d*, *J* = 6.8)	103.9
2‴	3.29 (1H, *dd*, *J*_1_ = 7.5, *J*_2_ = 9.8)	73.5	3.26 (1H, *dd*, *J*_1_ = 7.5, *J*_2_ = 9.0)	73.3	2‴	3.37 (1H, *dd*, *J*_1_ = 6.8, *J*_2_ = 8.3)	73.5
3‴	3.36 (1H, *dd*, *J*_1_ = 9.8, *J*_2_ = 9.8)	75.7	3.43 (1H, *dd*, *J*_1_ = 9.0, *J*_2_ = 9.0)	75.5	3‴	3.40 (1H, *dd*, *J*_1_ = 8.3, *J*_2_ = 8.7)	75.6
4‴	3.48 (1H, *ddd*, *J*_1_ = 5.3, *J*_2_ = 9.8, *J*_3_ = 10.2)	69.6	3.54 (1H, *ddd*, *J*_1_ = 5.7, *J*_2_ = 9.0, *J*_3_ = 9.6)	69.3	4‴	3.53 (1H, *ddd*, *J*_1_ = 4.9, *J*_2_ = 8.3, *J*_3_ = 8.7)	69.6
5‴	3.17 (1H, *dd*, *J*_1_ = 10.2, *J*_2_ = 11.7, H-5_a_‴)	65.2	3.19 (1H, *dd*, *J*_1_ = 9.6, *J*_2_ = 10.5, H-5_a_‴)	65.1	5‴	3.23 (1H, *dd*, *J*_1_ = 8.7, *J*_2_ = 11.3, H-5_a_‴)	65.2
	3.84 (1H, *dd*, *J*_1_ = 5.3, *J*_2_ = 11.7, H-5_e_‴)		3.83 (1H, *dd*, *J*_1_ = 5.7, *J*_2_ = 10.5, H-5_e_‴)			3.94 (1H, *dd*, *J*_1_ = 4.9, *J*_2_ = 11.3, H-5_e_‴)	

^a^ Data acquired with TMS as the internal standard, δ in ppm. Multiplicities and coupling constants (in Hz) are given in parentheses. Assignments confirmed by ^1^H-^1^H COSY, HMQC, and HMBC experiments. ^b^ For trivial atom numbering, see the chemical formula of DGQ (wintergreenoside A) and DGK (wintergreenoside B) (Figure 3). ^c^ Data acquired in methanol-*d*_4_. ^d^ Data acquired in water-*d*_2_.

**Table 2:** Anti-inflammatory and antioxidant activity of the selected compounds.

Analyte	Anti-Inflammatory Activity		Antioxidant Activity	
	COX-2	HYAL	O_2_^•−^	FRAP
	IC_50_ (mM) ^a^	IC_50_ (µM) ^a^	SC_50_ (µM) ^b^	mol Fe^2+^/mol ^c^
QU	1.56 ± 0.05 ^E^	101.84 ± 6.09 ^F^	25.08 ± 0.69 ^E^	14.23 ± 0.18 ^G^
MQ	1.29 ± 0.05 ^C^	98.15 ± 4.41 ^E,F^	32.55 ± 2.19 ^G^	9.24 ± 0.06 ^E^
DGQ	1.44 ± 0.06 ^D^	98.08 ± 2.31 ^E^	18.00 ± 1.26 ^D^	6.39 ± 0.14 ^D^
ECA	1.62 ± 0.07 ^E^	81.85 ± 3.96 ^D^	7.89 ± 0.41 ^C^	10.39 ± 0.27 ^F^
PB2	1.43 ± 0.06 ^D^	37.42 ± 1.78 ^B^	6.26 ± 0.09 ^B^	17.11 ± 0.06 ^I^
CB1	1.56 ± 0.07 ^E^	37.69 ± 0.80 ^B^	5.31 ± 0.12 ^A^	16.30 ± 0.10 ^H^
CHA	2.86 ± 0.13 ^F^	80.69 ± 3.42 ^D^	19.73 ± 0.42 ^D^	9.06 ± 0.18 ^E^
GT	0.78 ± 0.03 ^B^	64.02 ± 2.87 ^C^	1012.00 ± 31.72 ^I^	0.29 ± 0.02 ^A^
IND	0.50 ± 0.02 ^A^	35.69 ± 5.34 ^A^	-	-
DEX	1.29 ± 0.04 ^C^	36.13 ± 2.68 ^A,B^	-	-
TX	-	-	540.33 ± 4.04 ^H^	2.98 ± 0.06 ^B^
AA	-	-	29.87 ± 0.51 ^F^	3.97 ± 0.02 ^C^

^a^ IC_50_, half-maximal inhibitory concentration (amount of an analyte needed for 50% inhibition of enzyme activity); ^b^ SC_50_, half-maximal scavenging efficiency (amount of antioxidant needed to decrease the initial concentration of the oxidant by 50%); ^c^ antioxidant activity expressed in mol of ferrous ions (Fe^2+^) produced by 1 mol of an analyte. Analytes: QU, quercetin; MQ, miquelianin; DGQ, wintergreenoside A; ECA, (−)-epicatechin; PB2, procyanidin B2; CB1, cinnamtannin B-1; CHA, chlorogenic acid; GT, gaultherin. Positive controls: AA, ascorbic acid; DEX, dexamethasone; IND, indomethacin; TX, Trolox. Results presented as mean values ± SD (*n* = 3). For each parameter, different capital letters given in parentheses (A–I) indicate significant differences (*p* < 0.05).

**Table 3:** The optimised elution profile.

Time (min)	Solvent A (0.5% Aqueous Solution of Orthophosphoric Acid, *w*/*v*, %)	Solvent B (Acetonitrile, %)
0–1.0	94 (isocratic elution)	6 (isocratic elution)
1.0–8.5	94→86 (linear gradient)	6→14 (linear gradient)
8.5–15.0	86→84 (linear gradient)	14→16 (linear gradient)
15.0–23.0	84→50 (linear gradient)	16→50 (linear gradient)
23.0–24.0	50 (isocratic elution)	50 (isocratic elution)
24.0–25.0	50→94 (linear gradient, return to the initial conditions)	50→6 (linear gradient, return to the initial conditions)
25.0–30.0	94 (equilibration)	6 (equilibration)

**Table 4:** Validation data for the proposed HPLC-PDA method.

Analyte	t*_R_* ± SD (min)	*λ* (nm)	Linearity				Sensitivity	
			Linear Regression	*r*	Linear Range (μg/mL)	*F*-Test	LOD (μg/mL)	LOQ (µg/mL)
NCHA	3.86 ± 0.02	325	*y* = 45,336.38*x*	0.9992	0.37–122.4	5072.1	0.122	0.407
CHA	6.29 ± 0.02	325	*y* = 45,621.24*x*	0.9993	0.32–106.6	5430.2	0.107	0.357
CCHA	7.29 ± 0.02	325	*y* = 43,237.18*x*	0.9994	0.36–120.5	5753.8	0.121	0.403
PB2	8.20 ± 0.02	280	*y* = 14,435.01*x*	0.9993	0.85–56.6	9200.8	0.283	0.943
ECA	9.07 ± 0.02	280	*y* = 12,576.01*x*	0.9995	0.81–53.7	12,206.4	0.269	0.897
GT	9.51 ± 0.02	285	*y* = 4501.262*x*	0.9995	1.65–551.0	13,779.8	0.551	1.837
CB1	10.12 ± 0.02	280	*y* = 13,183.35*x*	0.9998	0.89–59.6	30,390.9	0.298	0.993
DGQ	12.69 ± 0.02	350	*y* = 25,690.20*x*	0.9990	0.83–55.4	6606.6	0.277	0.923
HY	14.52 ± 0.03	350	*y* = 29,715.40*x*	0.9994	0.85–56.5	10,507.6	0.283	0.943
MQ	15.51 ± 0.03	350	*y* = 32,178.87*x*	0.9995	0.64–84.8	13,826.4	0.212	0.707
QU	20.67 ± 0.01	370	*y* = 62,719.28*x*	0.9995	0.41–55.3	14,002.5	0.138	0.460

t*_R_*, retention time; *λ*, detection wavelength; *y*, peak area; *x*, concentration of standard in μg/mL; *F*-test, value of the statistical Fisher variance ratio for the experimental data. LOD, limit of detection. LOQ, limit of quantification. Analytes: QU, quercetin; MQ, miquelianin; HY, hyperoside; DGQ, wintergreenoside A; ECA, (−)-epicatechin; PB2, procyanidin B2; CB1, cinnamtannin B-1; NCHA, neochlorogenic acid; CHA, chlorogenic acid; CCHA, cryptochlorogenic acid; GT, gaultherin.

**Table 5:** Precision and accuracy data for the proposed HPLC-PDA method.

Analyte	Precision (RSD, %)				Accuracy
	Intra-Day Variability		Inter-Day Variability		Mean Recovery ± SD (%)
	t*_R_* (Retention Time)	Peak Area	t*_R_* (Retention Time)	Peak Area	
NCHA	0.41	0.39	1.72	3.47	99.45 ± 2.16
CHA	0.30	0.84	1.65	3.65	100.00 ± 3.25
CCHA	0.27	0.85	1.48	3.53	99.23 ± 3.33
PB2	0.28	1.58	1.78	3.88	99.15 ± 2.97
ECA	0.27	1.27	1.55	3.73	98.92 ± 2.55
GT	0.21	0.34	1.33	2.74	101.16 ± 3.46
CB1	0.32	1.93	1.47	3.76	98.78 ± 3.77
DGQ	0.24	1.32	1.35	3.85	99.86 ± 2.32
HY	0.26	1.53	1.54	3.77	99.91 ± 2.15
MQ	0.28	1.46	1.62	3.54	99.33 ± 2.73
QU	0.09	1.32	1.73	4.12	99.65 ± 2.49

Analytes: QU, quercetin; MQ, miquelianin; HY, hyperoside; DGQ, wintergreenoside A; ECA, (−)-epicatechin; PB2, procyanidin B2; CB1, cinnamtannin B-1; NCHA, neochlorogenic acid; CHA, chlorogenic acid; CCHA, cryptochlorogenic acid; GT, gaultherin.

## Data Availability

Not applicable.

## Supplementary Materials

The following are available online at https://www.mdpi.com/article/10.3390/ijms222111532/s1.

## Abbreviations

BF-AP	*n*-butanol fraction of MED-AP
CB1	cinnamtannin B-1
CCHA	cryptochlorogenic acid (4-*O*-caffeoylquinic acid)
CHA	chlorogenic acid (5-*O*-caffeoylquinic acid)
COX-2	cyclooxygenase 2
DEF-AP	diethyl ether fraction of MED-AP
DEX	dexamethasone
DGK	kaempferol 3-*O*-*β*-d-xylopyranosyl-(1→2)-*β*-d-glucuronopyranoside
DGQ	quercetin 3-*O*-*β*-d-xylopyranosyl-(1→2)-*β*-d-glucuronopyranoside
dw	dry weight
EAF-AP	ethyl acetate fraction of MED-AP
ECA	(−)-epicatechin
ELA-2	elastase 2
*f*MLP	*N*-formyl-l-methionyl-l-leucyl-l-phenylalanine
FRAP	ferric reducing antioxidant power
GV	quercetin 3-*O*-α-l-arabinopyranoside (guaijaverin)
GT	methyl salicylate 2-*O*-*β*-d-xylopyranosyl-(1→6)-*β*-d-glucuronopyranoside (gaultherin)
HY	quercetin 3-*O*-*β*-d-galactopyranoside (hyperoside)
HYAL	hyaluronidase
IL	interleukin
IND	indomethacin
IQ	quercetin 3-*O*-*β*-d-glucopyranoside (isoquercitrin)
KG	kaempferol 3-*O*-*β*-d-glucuronopyranoside
LPS	bacterial lipopolysaccharide
ME-AP	methanol-water (75:25, *v*/*v*) extract of aerial parts
MED-AP	defatted methanol-water (75:25, *v*/*v*) extract of aerial parts
ME-L	methanol-water (75:25, *v*/*v*) extract of leaves
ME-S	methanol-water (75:25, *v*/*v*) extract of stems
MQ	quercetin 3-*O*-*β*-d-glucuronopyranoside (miquelianin)
MQK	quercetin 3-*O*-*β*-d-glucuronopyranoside potassium salt
NCHA	neochlorogenic acid (3-*O*-caffeoylquinic acid)
O_2_^•−^	superoxide anion
PB2	procyanidin B2
QU	quercetin
ROS	reactive oxygen species
TNF-α	tumour necrosis factor α
WF-AP	water fraction of MED-AP
