# Supplementary material for: Screening for the Active Anti-Inflammatory and Antioxidant Polyphenols of Gaultheria procumbens and Their Application for Standardisation: From Identification through Cellular Studies to Quantitative Determination
Source: Int J Mol Sci. 2021 Oct 26;22(21):11532. doi: 10.3390/ijms222111532 (PMC8583782; doi:10.3390/ijms222111532)
Supplement: Supplementary file 1 [file ijms-22-11532-s001.zip › ijms-1408579-supplementary.pdf]

International Journal of *Molecular Sciences*

*Article*

# Screening for the Active Anti-Inflammatory and Antioxidant Polyphenols of *Gaultheria procumbens* and their Application for Standardisation: From Identification, through Cellular Studies to Quantitative Determination

**Monika Anna Olszewska [1], Aleksandra Owczarek [1], Anna Magiera [1], Sebastian Granica [2], and Piotr Michel [1,*]**

[1] Department of Pharmacognosy, Faculty of Pharmacy, Medical University of Lodz, Muszynskiego 1 St., 90-151 Lodz, Poland
[2] Microbiota Lab, Centre for Preclinical Studies, Department of Pharmacognosy and Molecular Basis of Phytotherapy, Medical University of Warsaw, 1 Banacha St., 02-097 Warsaw, Poland
* Correspondence: piotr.michel@umed.lodz.pl (P. Michel); Tel.: +48-426779169

## Supplementary Materials

**Table S1.** Phenolic analytes detected in *G. procumbens* aerial part dry extract by UHPLC-PDA-ESI-$MS^3$.

**Figure S1.** Proposed fragmentation scheme of cinnamtannin B-1 (CB1) in LC-ESI-$MS^3$.

**Table S2.** NMR spectral data of cinnamtannin B-1 (CB1) in methanol-$d_4$ (600 MHz for $^1$H NMR and 150.9 MHz for $^{13}$C NMR).

**Figure S2.** Effect of the selected compounds at 25-75 μM on viability (membrane integrity) of neutrophils as indicated by propidium iodide positive [PI(+)] cells.

**Table S3.** Content of the marker compounds in the dry extracts of *G. procumbens*.

**Table S1.** Phenolic analytes detected in *G. procumbens* aerial part dry extract by UHPLC-PDA-ESI-$MS^3$.

| Peak | Analyte | $t_R$ (min) | UV $\lambda_{max}$ (nm) | $[M–H]^-$ *m/z* | $MS^2$ (% relative abundance) | $MS^3$ (% relative abundance) |
|---|---|---|---|---|---|---|
| 1 | protocatechuic acid hexoside | 4.1 | 259, 293 | 315 | 225 (3); 153 (100); 107 (5) | |
| 2 | protocatechuic acid * | 4.7 | 259, 293 | 153 | | |
| 3 | protocatechuic acid hexoside | 5.8 | 259, 293 | 315 | 225 (1); 153 (100); 107 (2) | |
| 4 | 3-*O*-caffeoylquinic acid (neochlorogenic acid, NCHA) * | 6.5 | 325 | 353 | 191 (69); 179 (46) | |
| 5 | 3-*O*-*p*-coumaroylquinic acid derivative | 7.0 | 310 | 371 | 353 (12); 325 (100); 163 (73) | |
| 6 | procyanidin A-type dimer | 9.1 | 280 | 575 | 499 (73); 490 (100); 451 (18); 407 (2); 289 (35) | |
| 7 | procyanidin A-type dimer | 9.6 | 280 | 575 | 499 (79); 490 (100); 451 (30); 411 (13); 289 (21) | |
| 8 | procyanidin B-type dimer | 10.0 | 280 | 577 | **425 (100)**; 407 (49); 289 (3) | 407 (100); 381 (6); 341 (4); 273 (7) |
| 9 | procyanidin B-type dimer | 10.2 | 280 | 577 | **425 (100)**; 407 (56); 289 (18) | 407 (100); 381 (2); 273 (5) |
| 10 | unknown compound | 10.9 | 280 | 765 | 475 (40); 443 (24); 289 (100); 245 (8) | |
| 11 | 5-*O*-caffeoylquinic acid (chlorogenic acid, CHA) * | 11.4 | 325 | 353 | 191 (100); 179 (3) | |
| 12 | methyl salicylate 2-*O*-*β*-D-glucopyranosyl-(1→2)-*β*-D-glucopyranoside (physanguloside A) * | 12.2 | 285 | 521 *** | 475 (29); 443 (3); 329 (100) | |
| 13 | methyl salicylate 2-*O*-*β*-D-glucopyranosyl-(1→2)-[*O*-*β*-D-xylopyranosyl-(1→6)]-*O*-*β*-D-glucopyranoside * | 12.6 | 285 | 653 *** | 607 (100); 575 (11) | |
| 14 | 4-*O*-caffeoylquinic acid (cryptochlorogenic acid, CCHA) * | 12.8 | 325 | 353 | 173 (100) | |
| 15 | procyanidin B-type dimer | 14.0 | 280 | 577 | **425 (100)**; 407 (64); 289 (8) | 407 (100); 381 (5); 273 (8) |
| 16 | procyanidin B2 (PB2) ** | 15.2 | 280 | 577 | **425 (100)**; 407 (51); 289 (15) | 407 (100); 273 (6) |
| 17 | procyanidin B-type trimer | 15.5 | 280 | 865 | 847 (17); 739 (76); **713 (37)**; 695 (77); 577 (78); 451 (25); 287 (25) | 695 (100); 575 (47); 561 (9); 425 (28); 405 (43); 287 (15) |
| 18 | gaultherin isomer | 15.7 | 285 | 491 *** | 445 (7); 413 (10); 293 (100); 149 (4) | |
| 19 | (–)-epicatechin (ECA) ** | 16.5 | 280 | 289 | 245 (100); 205 (23) | |
| 20 | gaultherin isomer (GT) ** | 17.1 | 285 | 491 *** | 445 (7); 413 (4); 293 (100); 149 (2) | |
| 21 | unknown compound | 17.7 | 280 | 363 | 345 (4); 183 (100); 179 (19); 143 (7); 121 (14) | |
| 22 | procyanidin A-type trimer | 18.4 | 280 | 863 | **711 (100)**; 573 (34); 559 (17); 451 (47); 411 (79); 289 (100) | 693 (100); 559 (54); 541 (16); 407 (17) |

**Table S1.** *Cont.*

| Peak | Analyte | $t_R$ (min) | UV $\lambda_{max}$ (nm) | [M–H]⁻ *m/z* | MS² (% relative abundance) | MS³ (% relative abundance) |
|---|---|---|---|---|---|---|
| 23 | procyanidin A-type trimer (cinnamtannin B-1, CB1) ** | 20.0 | 280 | 863 | **711 (100)**; 693 (24); 573 (23); 559 (21); 451 (33); 411 (42); 289 (11) | 693 (88); 559 (100); 541 (8); 463 (3); 407 (6); 285 (1) |
| 24 | procyanidin B-type trimer (procyanidin C1) * | 21.2 | 280 | 865 | 847 (6); 739 (42); **713 (43)**; 695 (100); 577 (80); 451 (31); 287 (59) | 695 (100); 575 (39); 561 (89); 407 (23); 243 (9) |
| 25 | procyanidin A-type trimer | 21.6 | 280 | 863 | **711 (100)**; 573 (43); 559 (16); 451 (45); 411 (70); 289 (20) | 693 (100); 559 (69); 541 (22); 463 (24); 407 (58) |
| 26 | caffeoylquinic acid derivative | 22.0 | 325 | 391 | 225 (26); 179 (100); 161 (12); 143 (7); 119 (8); 113 (5) | |
| 27 | procyanidin A-type dimer | 22.5 | 280 | 575 | 499 (100); 491 (28); 451 (8); 411 (9); 289 (37) | |
| 28 | procyanidin A-type dimer | 23.4 | 280 | 575 | 499 (57); 491 (38); 451 (11); 411 (9); 289 (95) | |
| 29 | procyanidin A-type trimer | 24.2 | 280 | 863 | **711 (100)**; 573 (49); 559 (16); 451 (35); 411 (64); 289 (21) | 693 (100); 559 (65); 541 (20); 463 (21); 407 (55) |
| 30 | procyanidin A-type dimer | 24.7 | 280 | 575 | 499 (18); 491 (23); 451 (43); 411 (14); 289 (26) | |
| 31 | procyanidin A-type trimer | 25.1 | 280 | 863 | **711 (100)**; 573 (68); 559 (19); 451 (6); 411 (11); 289 (11) | 693 (100); 559 (45); 541 (28); 463 (27); 407 (50) |
| 32 | quercetin 3-*O*-*β*-D-xylopyranosyl-(1→2)-*β*-D-glucuronopyranoside (wintergreenoside A, DGQ) ** | 25.5 | 257, 356 | 609 | 477 (2); **301 (100)** | 273 (51); 255 (22); 179 (100); 151 (69) |
| 33 | procyanidin A-type dimer | 26.4 | 280 | 575 | 499 (30); 491 (19); 451 (12); 411 (39); 289 (100) | |
| 34 | procyanidin A-type trimer | 26.8 | 280 | 863 | **711 (100)**; 573 (60); 559 (11); 451 (35); 411 (92); 289 (39) | 693 (29); 559 (11); 541 (12); 463 (38); 407 (21) |
| 35 | quercetin 3-*O*-*β*-D-galactopyranoside (hyperoside, HY) ** | 27.6 | 254, 353 | 463 | **301 (100)** | 273 (49); 255 (19); 179 (95); 151 (75) |
| 36 | quercetin 3-*O*-*β*-D-glucopyranoside (isoquercitrin, IQ) ** | 28.5 | 256, 353 | 463 | **301 (100)** | 273 (54); 255 (22); 179 (100); 151 (76) |
| 37 | quercetin 3-*O*-*β*-D-glucuronopyranoside (miquelianin, MQ) ** | 29.1 | 256, 356 | 477 | **301 (100)** | 273 (29); 257 (19); 179 (100); 151 (58) |
| 38 | kaempferol 3-*O*-*β*-D-xylopyranosyl-(1→2)-*β*-D-glucuronopyranoside (wintergreenoside B, DGK) ** | 29.6 | 275, 345 | 593 | 461 (3); **285 (100)** | 267 (32); 257 (100); 241 (23); 229 (30); 151 (16) |
| 39 | quercetin 3-*O*-*α*-L-arabinopyranoside (guaijaverin, GV) ** | 30.8 | 258, 356 | 433 | **301 (100)** | 273 (44); 255 (21); 179 (100); 151 (68) |
| 40 | kaempferol 3-*O*-*β*-D-glucuronopyranoside (KG) ** | 33.5 | 265, 345 | 461 | **285 (100)** | 267 (28); 257 (100); 241 (15); 229 (45); 151 (22) |
| 41 | quercetin (QU) * | 43.7 | 255, 364 | 301 | 273 (41); 255 (18); 179 (100); 151 (74) | |

$t_R$, retention times; UV $\lambda_{max}$, absorbance maxima in PDA spectra; [M−H]⁻, pseudomolecular ions in MS spectra recorded in a negative ion mode; in bold – ions subjected to MS³ fragmentation; * compounds identified with authentic standards; ** compounds isolated during the present study; *** [M+HCOO]⁻. The nomenclature of caffeoylquinic acids isomers is according to IUPAC.

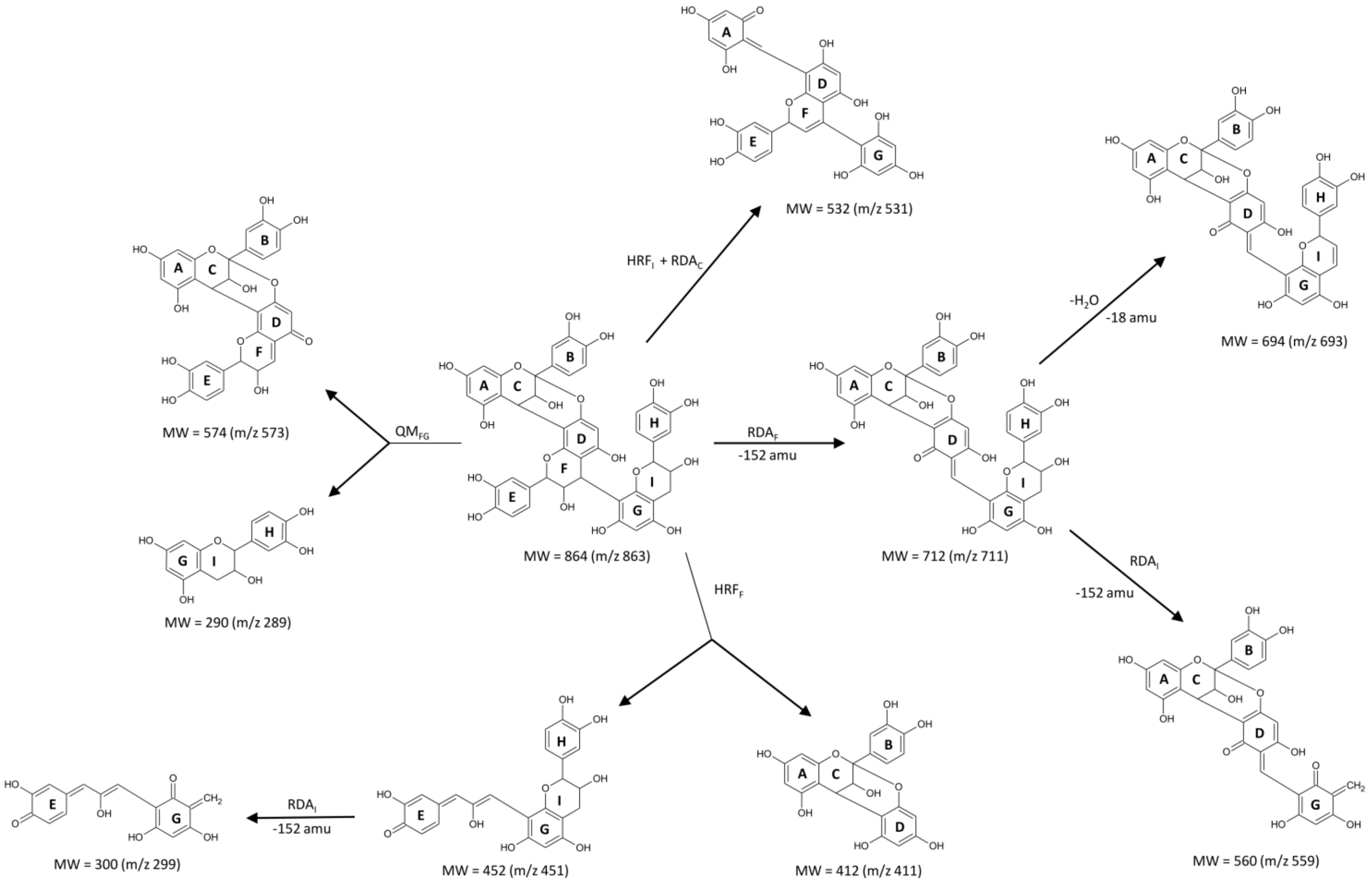


**Figure S1.** Proposed fragmentation scheme of cinnamtannin B-1 (CB1) in LC-ESI-MS$^3$. MW, molecular weight of a ion; HRF, heterocyclic ring fission; QM, quinone methide cleavage; RDA, retro-Diels-Alder fragmentation.

**Table S2.** NMR spectral data of cinnamtannin B-1 (CB1) in methanol-$d_4$ (600 MHz for $^1$H NMR and 150.9 MHz for $^{13}$C NMR) [a].

| Ring | Pos. [b] | $\delta_H$ | $\delta_C$ |
|---|---|---|---|
| C | 2 | | 101.2 |
| | 3 | 3.34 (1H, *d*, *J*=3.4) | 68.2 |
| | 4 | 4.19 (1H, *d*, *J*=3.4) | 29.9 |
| A | 5 | | 157.8 |
| | 6 | 6.01 (1H, *d*, *J*=2.3) | 99.4 |
| | 7 | | 158.9 |
| | 8 | 6.06 (1H, *d*, *J*=2.3) | 97.6 |
| | 9 | | 155.2 |
| | 10 | | 105.9 |
| B | 1' | | 133.5 |
| | 2' | 7.08 (1H, *d*, *J*=1.9) | 117.2 |
| | 3' | | 146.8 |
| | 4' | | 146.9 |
| | 5' | 6.88 (1H, *d*, *J*=8.3) | 117.1 |
| | 6' | 6.90 (1H, *dd*, $J_1$=2.3, $J_2$=8.3) | 120.9 |
| F | 2 | 5.74 (1H, *s*) | 79.9 |
| | 3 | 4.17 (1H, *br s*) | 73.6 |
| | 4 | 4.6 (1H, *br s*) | 39.3 |
| D | 5 | | 156.8 |
| | 6 | 5.85 (1H, *s*) | 97.2 |
| | 7 | | 152.1 |
| | 8 | | 107.5 |
| | 9 | | 152.8 |
| | 10 | | 107.8 |
| E | 1' | | 132.8 |
| | 2' | 7.35 (1H, *d*, *J*=1.5) | 117.8 |
| | 3' | | 147.3 |
| | 4' | | 147.6 |
| | 5' | 6.83 (1H, *d*, *J*=8.3) | 116.8 |
| | 6' | 7.22 (1H, *dd*, $J_1$=1.5, $J_2$=8.3) | 122.4 |
| I | 2 | 4.43 (1H, *s*) | 81.4 |
| | 3 | 3.91 (1H, *br s*) | 68.6 |
| | 4 | 2.88 (2H, *m*) | 30.9 |
| G | 5 | | 157.1 |
| | 6 | 6.15 (1H, *s*) | 97.6 |
| | 7 | | 156.6 |
| | 8 | | 109.9 |
| | 9 | | 156.8 |
| | 10 | | 100.9 |
| H | 1' | | 134.2 |
| | 2' | 6.86 (1H, *d*, *J*=1.5) | 116.4 |
| | 3' | | 146.4 |
| | 4' | | 146.5 |
| | 5' | 6.79 (1H, *d*, *J*=8.3) | 116.5 |
| | 6' | 6.76 (1H, *dd*, $J_1$=1.5, $J_2$=8.3) | 120.6 |

[a] Data acquired with TMS as the internal standard, δ in ppm. Multiplicities and coupling constants (*J*, in Hz) are given in parentheses. Assignments confirmed by $^1$H-$^1$H COSY, HMQC, HMBC and ROESY experiments. [b] For trivial atom numbering see chemical formula of CB1 (Fig. 2).

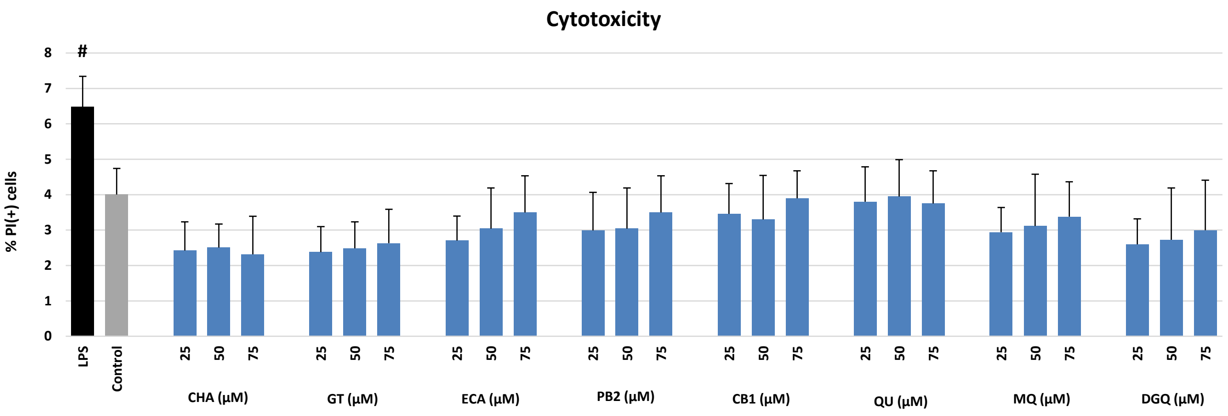


**Figure S2.** Effect of the selected compounds at 25-75 µM on viability (membrane integrity) of neutrophils as indicated by propidium iodide positive [PI(+)] cells. Data expressed as means ± SD of three independent experiments performed with cells isolated from five independent donors. Statistical significance: #$p < 0.05$ compared to the non-stimulated control. Analytes: QU, quercetin; MQ, miquelianin; DGQ, wintergreenoside A; ECA, (–)-epicatechin; PB2, procyanidin B2; CB1, cinnamtannin B-1; CHA, chlorogenic acid; GT, gaultherin.

**Table S3.** Content of the marker compounds in the dry extracts of *G. procumbens*.

| Analyte | Content (mg/g dw) [a] | | | | | | | |
|---|---|---|---|---|---|---|---|---|
| | ME-AP | ME-L | ME-S | MED-AP | DEF-AP | EAF-AP | BF-AP | WF-AP |
| NCHA | 3.68 ± 0.13 D | 4.24 ± 0.08 E | 0.96 ± 0.04 A | 2.48 ± 0.15 C | 1.67 ± 0.06 B | 5.68 ± 0.22 F | 6.47 ± 0.06 G | 2.34 ± 0.15 C |
| CHA | 1.25 ± 0.09 C | 1.68 ± 0.11 D | 0.96 ± 0.05 B | 1.67 ± 0.10 D | 2.46 ± 0.13 F | 2.72 ± 0.13 G | 2.04 ± 0.08 E | 0.41 ± 0.01 A |
| CCHA | 0.65 ± 0.03 A | 0.88 ± 0.10 B | *nd* | 1.95 ± 0.11 D | 0.81 ± 0.05 B | 3.78 ± 0.22 E | 3.56 ± 0.20 E | 1.24 ± 0.03 C |
| PB2 | 14.25 ± 0.45 E | 13.15 ± 0.37 D | 17.22 ± 0.19 D | 13.46 ± 0.25 D | 6.12 ± 0.55 B | 35.05 ± 0.65 F | 8.36 ± 0.21 C | 2.10 ± 0.07 A |
| ECA | 12.47 ± 0.67 C | 9.07 ± 0.29 B | 24.35 ± 0.93 D | 12.31 ± 0.16 C | 0.31 ± 0.07 A | 79.73 ± 1.58 E | *nd* | *nd* |
| GT | 96.51 ± 0.80 F | 98.41 ± 0.31 E | 93.76 ± 0.81 D | 75.68 ± 0.80 B | 21.37 ± 1.11 A | 30.25 ± 1.47 C | 127.69 ± 4.06 G | 27.81 ± 1.99 B |
| CB1 | 19.84 ± 0.22 C | 18.61 ± 0.28 B | 25.11 ± 0.52 D | 14.42 ± 0.22 A | *nd* | 154.57 ± 4.52 E | 15.51 ± 0.78 A | *nd* |
| DGQ | 5.13 ± 0.38 E | 7.26 ± 0.24 F | 2.16 ± 0.14 C | 3.90 ± 0.38 D | 1.02 ± 0.04 B | 0.40 ± 0.01 A | 5.05 ± 0.09 E | 10.65 ± 0.52 G |
| HY | 3.24 ± 0.25 B | 5.01 ± 0.16 D | 1.04 ± 0.03 A | 5.34 ± 0.25 D | 4.30 ± 0.17 C | 30.04 ± 1.69 F | 9.88 ± 0.08 E | *nd* |
| MQ | 27.75 ± 0.78 D | 32.14 ± 0.69 E | 17.54 ± 0.57 B | 30.38 ± 0.78 C | 9.72 ± 0.55 A | 36.90 ± 1.34 F | 42.74 ± 0.24 G | 22.84 ± 0.88 C |
| QU | 0.19 ± 0.01 C | 0.22 ± 0.01 C | 0.1 ± 0.01 B | 0.48 ± 0.01 D | 8.68 ± 0.46 F | 5.36 ± 0.18 E | 7.74 ± 0.29 G | 0.07 ± 0.01 A |
| Total | 184.96 | 190.67 | 181.04 | 162.07 | 56.46 | 384.48 | 229.04 | 67.46 |

Analytes: QU, quercetin; MQ, miquelianin; HY, hyperoside; DGQ, wintergreenoside A; ECA, (–)-epicatechin; PB2, procyanidin B2; CB1, cinnamtannin B-1; NCHA, neochlorogenic acid; CHA, chlorogenic acid; CCHA, cryptochlorogenic acid; GT, gaultherin. Extracts: methanol-water (75:25, *v*/*v*) extracts (ME) of the aerial parts (ME-AP), leaves (ME-L), and stems (ME-S); deffated methanol-water (75:25, *v*/*v*) extract of aerial parts (MED-AP), and its fractions: DEF-AP (diethyl ether fraction), EAF-AP (ethyl acetate fraction), BF-AP (*n*-butanol fraction), and WF-AP (water fraction). All data expressed as mean values ± SD ($n$ = 3 × 3 × 1); [a] dry weight of the plant extract; different superscripts (A-G) indicate significant differences in the mean value at $\alpha$ = 0.05; *nd* – not detected.
